# Eradication of Mature Bacterial Biofilms with Concurrent Improvement in Chronic Wound Healing Using Silver Nanoparticle Hydrogel Treatment

**DOI:** 10.3390/biomedicines9091182

**Published:** 2021-09-08

**Authors:** Hanif Haidari, Richard Bright, Sanjay Garg, Krasimir Vasilev, Allison J. Cowin, Zlatko Kopecki

**Affiliations:** 1 Clinical & Health Sciences, University of South Australia, Adelaide, SA 5000, Australia; Hanif.haidari@unisa.edu.au (H.H.); Sanjay.garg@unisa.edu.au (S.G.); 2 Future Industries Institute, University of South Australia, Mawson Lakes, SA 5095, Australia; Krasimir.vasilev@unisa.edu.au; 3 Academic Unit of STEM, University of South Australia, Mawson Lakes, SA 5095, Australia; Richard.bright@unisa.edu.au

**Keywords:** wound infection, antibiofilm hydrogel, AgNP hydrogel, stimuli responsive hydrogel, wound healing, controlled release, wound biofilm eradication, mature biofilm

## Abstract

Biofilm-associated infections are a major cause of impaired wound healing. Despite the broad spectrum of anti-bacterial benefits provided by silver nanoparticles (AgNPs), these materials still cause controversy due to cytotoxicity and a lack of efficacy against mature biofilms. Herein, highly potent ultrasmall AgNPs were combined with a biocompatible hydrogel with integrated synergistic functionalities to facilitate elimination of clinically relevant mature biofilms in-vivo combined with improved wound healing capacity. The delivery platform showed a superior release mechanism, reflected by high biocompatibility, hemocompatibility, and extended antibacterial efficacy. In vivo studies using the *S. aureus* wound biofilm model showed that the AgNP hydrogel (200 µg/g) was highly effective in eliminating biofilm infection and promoting wound repair compared to the controls, including silver sulfadiazine (Ag SD). Treatment of infected wounds with the AgNP hydrogel resulted in faster wound closure (46% closure compared to 20% for Ag SD) and accelerated wound re-epithelization (60% for AgNP), as well as improved early collagen deposition. The AgNP hydrogel did not show any toxicity to tissue and/or organs. These findings suggest that the developed AgNP hydrogel has the potential to be a safe wound treatment capable of eliminating infection and providing a safe yet effective strategy for the treatment of infected wounds.

## 1. Introduction

Wound infection is a major medical threat as continuous colonization of pathogens impairs healing and can leads to sepsis [1,2]. Chronic wounds provide a fertile environment for the development of mature biofilms, which offer bacteria protection from antibiotics or host defence proteins [3,4]. *Staphylococcus aureus* (*S. aureus*) bacteria has been identified as a leading cause of wound infection with the ability to develop multiple-antibiotic resistance and lead to chronic infections that are hard to combat [5]. Application of higher doses of therapeutic agents is often required for the complete elimination of pathogens because the physical and metabolic barriers provided by biofilms lead to high resistance and poor penetrations of antimicrobials [6]. Moreover, chronic wounds associated with biofilm infection are difficult to heal with subsequent effects on tissue functionality and scarring [7]. Therefore, treatment strategies aimed at the mitigation of bacterial biofilms with the capacity to promote healing have attracted significant attention.

Silver nanoparticles (AgNPs) that have antibiofilm activity, including multimodal efficacy in biofilm pore-forming mechanism and broad-spectrum activity, are regarded as a promising approach to tackle mature biofilm wound infections [8,9]. The biofilm penetration functionality is largely dependent on nanoparticle size, surface charge, and stability with smaller-sized AgNPs showing greater uptake and availability at the site of infection [10]. However, the success of AgNPs in treating wound infections has been constrained, primarily due to their toxicity toward mammalian cells, uncontrolled release of silver ions (Ag^+^), and susceptibility aggregation in the wound environment, requiring significantly higher concentration for efficacy and often inconsistent bactericidal effects [11,12]. Additionally, the lack of widely available spectroscopic and microscopic real-time approaches to study the interaction between nanoparticles and cells in complex biological system to assess their efficacy and safety further prevents the therapeutic developments of AgNPs [13,14]. The clinical use of currently available silver-based dressings remains controversial due to stability and toxicity problems, as well as microbial resistance development [15].

To overcome these limitations, different types of silver formulations have been developed to enhance performance with emphasis on stability and antibacterial effects [16]. To enhance the antibacterial effect, AgNP hydrogels are often combined with antibiotics to endow greater bactericidal activity; however, the use of antibiotic-free systems is preferred to avoid resistance development [17]. The controlled release of Ag^+^ from a hydrogel is another important aspect, as studies have shown that AgNPs have a short lifetime (3 h) while the inconsistent and fast release of Ag^+^ contributes to high wound toxicity [18]. Consequently, the controlled release mechanism of AgNPs has been integrated with different delivery platforms i.e., antibacterial coatings [19], stimuli-responsive hydrogel to provide on-demand release [20], mussel-inspired hydrogels [21], and mesoporous silica nanoparticles with the slow dissolution of Ag^+^ [22], many of which show promising in-vitro results. While their antibacterial activity is relatively well studied using in-vitro models, the AgNPs’ efficacy against mature biofilms in clinically relevant animal models is unconvincingly reported. Additionally, studies to date have shown that AgNP hydrogels suffer salt instability and rapid aggregation compromising AgNPs’ ability to penetrate biofilms and leading to high cytotoxicity [23]. Therefore, a better understanding of AgNPs’ design and stability is critical for safety, efficacy, and clinical translation. The use of multifunctional materials in AgNP hydrogel design that combine antibacterial and healing promoting properties hold promise for successful clinical regeneration of infected wounds.

Our previous study demonstrated the potential of a thermoresponsive ultrasmall AgNP hydrogel as an antibacterial agent against acute wound infection (resembling planktonic bacterial growth) [24]. Most acute wound infections are transient and resolve spontaneously within 12 weeks depending on host immune responses [25]. However, chronic infections observed in non-healing wounds are usually associated with persistent bacterial colonization accompanied with delayed healing, which often requires long-term antibacterial therapy at a much higher concentration [26]. Despite the benefits of current antibacterial hydrogels to control short-term acute infection [24], the potential of these approaches has not been demonstrated in complex models of mature biofilm wound infection. These biofilm models feature higher levels of pathogenic bacteria—a larger wound size has a high degree of exudate and maceration and they utilize the application of a wound dressing, which not only closely mimics the clinical situation but also promotes mature biofilm development. The treatment of chronic wound infection often requires higher concentrations of antimicrobial agents, which may benefit by rapid killing but also result in mammalian cell toxicity [27]. Unregulated release of Ag^+^ results in non-selective killing [28] while the use of large size NPs requires higher silver concentrations to obtain efficacy [29]. One strategy to overcome these limitations is the fabrication of a controlled release system containing a stable and optimized concentration of ultrasmall AgNPs, harnessing high bactericidal effects with significantly lower or absent cytotoxicity. This tailored approach has been shown to be effective against planktonic bacteria [24]; however, its ability to overcome resistant mature biofilms in chronic wounds has not yet been reported.

**Scheme 1:**
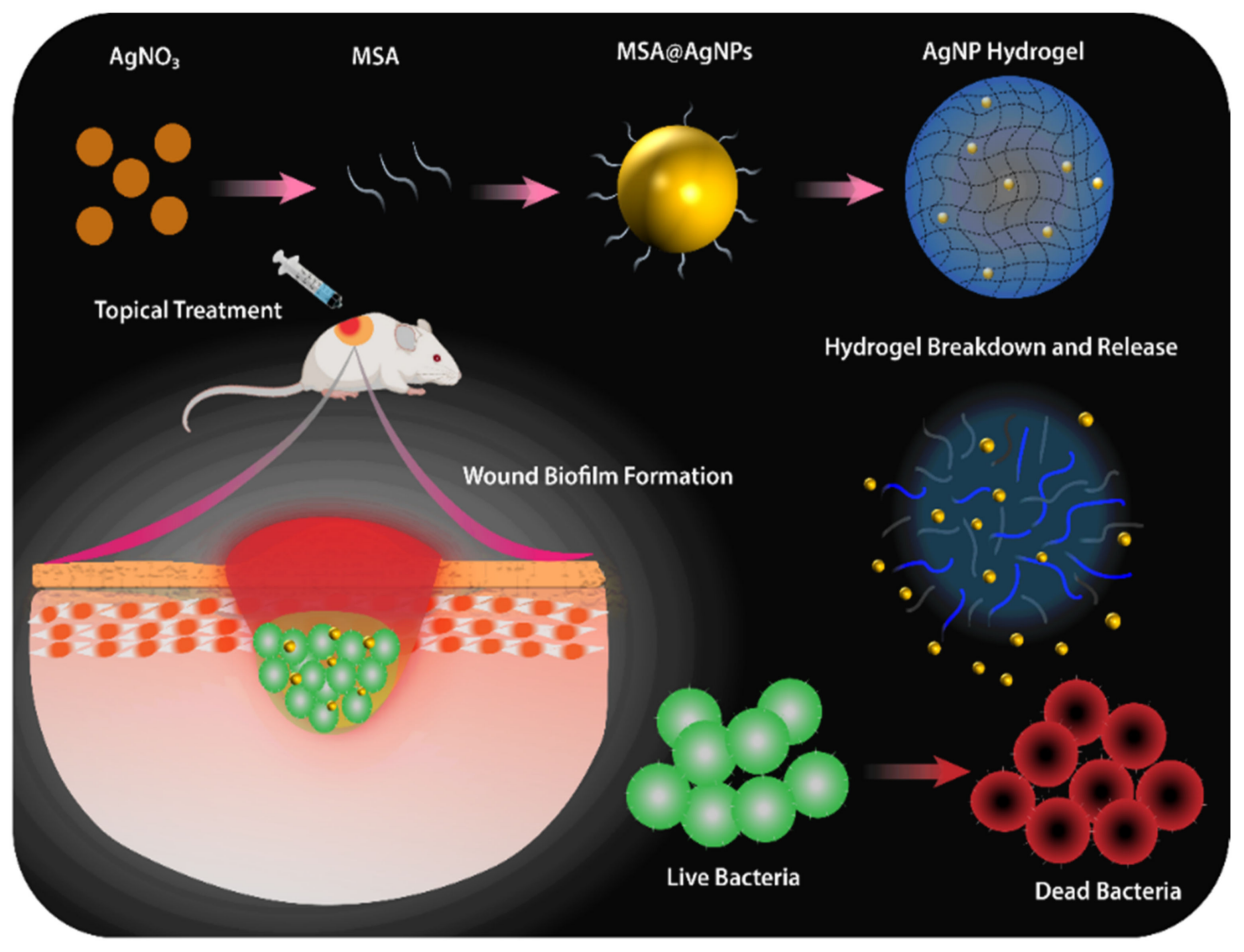
Schematic illustration of AgNP hydrogel formation and treatment of bacterial wound biofilms.

This study investigated the efficacy of a controlled release system of ultrasmall AgNPs against mature and clinically relevant *S. aureus* biofilms using a validated model of mature biofilm wound infection. Utilizing the inherent nature of water-soluble polymeric hydrogel as a tissue scaffold and integrated thermoresponsive functionality as a controlled release mechanism of ultrasmall AgNPs [30,31], for the first time, we demonstrated the ability of topically administered AgNP hydrogel to clear mature *S. aureus* biofilms and promote wound healing simultaneously (Scheme 1). The described AgNP hydrogel provides a versatile platform with features of antibacterial efficacy, exudate absorbance, low cost, biocompatibility, hemocompatibility, and improved wound healing, holding a great promise for future clinical translation.

## 2. Materials and Methods

### 2.1. Materials

All chemicals are commercially available and used without further purifications. AgNO_3_, mercaptosuccinic acid (MSA), pluronic f-127 (MW ~ 12,600 g/mol), propylene glycol (PG), resazurin sodium salt, sodium hydroxide (NaOH), sodium borohydride (NaBH_4_), were purchased from (Sigma Aldrich, Sydney, Australia). Dulbecco’s Modified Eagle Medium (DMEM), fetal calf serum (FCS), and phosphate buffered saline (PBS) were purchased from Gibco Life Technologies. Tryptic soy broth (TSB) and tryptic soy agar (TSA) was purchased from Thermo Fisher Scientific, Oxoid, and prepared according to the manufacturer’s instructions. Silver sulfadiazine (Ag SD) (Flamazine Cream (1%) 50 g) (Smith & Nephew, Sydney, Australia) was purchased from the pharmacy without further modifications. The Ag SD contains (10 mg/g) silver sulfadiazine, equivalent to 3.02 mg/g of Ag.

### 2.2. Preparation and Characterization of the AgNP Hydrogel

AgNPs were synthesized according to previously published protocol [32]. Briefly, 1 mL of 20 mM silver nitrate (AgNO_3_) and 1 mL of 20 mM mercaptosuccinic acid (MSA) were mixed in 7.7 mL of MilliQ water under continuous stirring (500 rpm) to form MSA–Ag complex. The 0.05 M sodium borohydride (NaBH_4_) acting as the reducing agent was added to the reaction with continued stirring for a further 24 h at ambient temperature to form MSA–AgNPs. After the synthesis, the AgNP solution were purified through dialysis (Pur-A-Lyzer™ Maxi Dialysis Kit; MWCO 3.5 kDa, Perth, Australia) against ultrapure water to remove impurities and excess precursors (i.e., NaBH_4_). The synthesized AgNP solution was characterized using UV-vis spectroscopy, dynamic light scattering (DLS), and transmission electron microscopy (TEM) (JEOL JEM-2100F-HR, Tokyo, Japan). The colloidal stability and primary size distribution of AgNP were confirmed before proceeding to hydrogel synthesis. Subsequently, the AgNP hydrogel was prepared by mixing a solution of AgNP into synthesizing pluronic f-127 (19%) hydrogel via continuous stirring under cold conditions until complete dissolution and distribution of hydrogel components [33]. The final components of the AgNP hydrogel are reported in Table S1. The AgNP hydrogel contained 200 µg/g of AgNPs while the control hydrogel (blank hydrogel) was prepared without AgNP. Both formulations were stored at 4 °C for further use. The interior morphology of hydrogels was characterized using scanning electron microscopy (SEM Carl Zeiss Crossbeam 540 with SDD EDS, Oberkochen, Germany, while the Rheometer (TA Instrument, DE, USA was used to monitor the gelation point of AgNP hydrogel in response to temperature changes (4–40 °C) and simultaneously measure the viscoelastic behavior and shear stress [33].

### 2.3. Stability and Release Kinetics of the AgNP Hydrogel

The stability of the AgNP was measured using dynamic light scattering (DLS) (Malvern Zetasizer Nano, Worcestershire, United Kingdom, including assessment in biological media (DMEM, supplemented with 10% FCS) to monitor the changes in the hydrodynamic diameter of NPs after 24 h incubation at 37 °C, simulating the physiological system. The in-vitro release of AgNPs from the hydrogel was investigated using a dialysis membrane (Dialysis 12Kda CelluSep, Perth, Australia) [34]. Briefly, the AgNP hydrogel sample (2 mL) was dispensed in the dialysis membrane containing 10 mL PBS as the release medium. The system was gently stirred at 100 rpm. Aliquots were withdrawn at predetermined time intervals (0–24 h) and replaced by the same volume of fresh solution. The samples were digested with nitric acid and Ag content was determined using inductively coupled plasma–optical emission spectrometry (ICP–OES) [34].

### 2.4. Bacterial Bioluminescent Strain and Growth

The in-vitro and in-vivo antibacterial experiments were undertaken using bioluminescent *S. aureus* (ATCC12600) carrying a modified lux operon from Photorhabdus luminescence (Xen 29) (PerkinElmer, Beaconsfield, United Kingdom). A stock of *S. aureus* (Xen 29) was obtained from (−80 °C) and streaked across tryptic soy agar (TSA) supplemented with kanamycin (200 µg/mL) to obtain pure isolated *S. aureus* (Xen 29) colonies. Thereafter, a single bacterial colony was sub-cultured in tryptic soy broth (TSB) supplemented with 200 µg/mL kanamycin and grown at 37 °C overnight. The optical density was measured at 600 nm (OD600) and standardized by diluting the overnight culture to 0.5, which equates to approximately 5 × 10^8^ CFU/mL. The resulting standardized inoculum was centrifuged, washed twice in PBS, and resuspended to the required density for wound inoculation, while maintaining an aseptic technique in the process.

### 2.5. In Vitro Cytocompatibility

Cell viability was determined using the resazurin assay and Live/Dead Mammalian™ viability kit (Invitrogen, Thermo Fisher Scientific, Sydney, Australia). Human foreskin fibroblasts (HFFs) were seeded in 96-well tissue culture plates at a density of 3 × 10^4^ cells/well or 24-well tissue culture plate on glass coverslip (1 × 10^5^ cells/well) in Dulbecco’s modified Eagle’s medium (DMEM), supplemented with 10% fetal calf serum (FCS) and 5% penicillin and streptomycin and incubated at 37 °C in 5% CO_2_ for 24 h. Extracts of the AgNP hydrogel were prepared similar to a previously reported method [33]. After cell confluency, the wells were washed with PBS and treated with hydrogel extracts. Cells treated with fresh DMEM were used as controls. After further incubation for 24 h, the cells were washed and resazurin dye was added and fluorescence intensity was measured after 2 h incubation at 37 °C. Cells that were grown and treated in glass coverslips were stained with Live/Dead staining according to the manufacturers’ instructions, similar to our previous method [20]. Samples were imaged via Olympus IX81 epifluorescence microscope (Olympus, Tokyo, Japan).

### 2.6. Hemolytic Activity of the Test Hydrogels 

Blood was collected from mice using terminal cardiac bleed protocol following approval from UniSA Animal Ethics Committee for scavenging tissue from excess breeding mice at Centralized Animal Facility UniSA following approved protocols. Using mice blood, the red blood cells (RBCs) were isolated by centrifugation at (1000 rpm) for 10 min, and the hemolysis was measured similar to previous published protocol with slight modifications [35]. The obtained erythrocytes were washed three times with PBS and then diluted to a final concentration of 5% (*v*/*v*). The 500 µL of the hydrogel test samples were dispersed into an equal volume of RBCs suspension (5%) in a 2 mL centrifuge tube and incubated at 37 °C for 1 h with moderate shaking (120 rpm). Samples were then further centrifuged at (1000 rpm) for 10 min and the supernatants (100 µL) were added into a 96-well plate for measuring the absorbance. The absorbance of the solution was measured using a microplate reader (FLOUstar Optima, BMG Labtech, Melbourne, Australia) at 540 nm. Here the water served as the negative control while the 0.1% Triton X-100 served as the positive control. The percentage of hemolysis of each sample was calculated using the following equation, hemolysis % = (Test sample − Neg control)/(Pos control − Neg control) × 100.

### 2.7. In Vitro Anti-Biofilm Eradication of AgNP Hydrogel 

The in vitro antibiofilm activity of the AgNP hydrogel was evaluated using the Live/Dead Baclight™ viability kit (Invitrogen, Thermo Fisher Scientific, Sydney, Australia) [33]. *S. aureus* (Xen 29) cells were selectively grown overnight with kanamycin to mid-log phase. The overnight bacterial culture was then adjusted to (2 × 10^6^ CFU/mL) and added to wells containing sterile glass coverslips in a 24-well plate. The plate was statically incubated at 37 °C for 24 h to allow biofilm formation. The planktonic cells were gently washed, and the biofilms were treated with 400 μL of hydrogel extracts together with silver sulfadiazine and the negative control [33]. The final concentration of AgNPs in each well was 40 µg compared to 600.4 µg of silver for the Ag SD group. After a further 24 h of incubation at 37 °C, the coverslips were washed and then stained with 3 μm of SYTO9 and propidium iodide (PI) followed by 15 min in the dark at 25 °C. The excess stain was washed and mounted on a glass slide for confocal imaging. Images were visualized under CLSM and processed with Bitplane Imaris v9.0 3D/4D image analysis software.

### 2.8. In Vivo Mature Biofilm Wound Infection Animal Model and Treatment 

All mice (BALB/C wildtype, M/F 10–12 weeks old, 18–20 g) were obtained from the Animal Resources Centre (ARC, Perth, Australia). All animal experiments were conducted in compliance with the guidelines for the care and use of research animals established by the University of South Australia Animal Ethics committee UniSA AEC (U39-19). After allowing 7 days to acclimatize, male and female mice were randomized into three groups, each group containing (*n* = 8): the blank hydrogel, Ag SD, and AgNP hydrogel. The pre-surgery anaesthesia was induced by 2% isoflurane in 1.5/L of O_2_. The backs of the mice were shaved, cleaned, and disinfected before surgery. Pre-operative analgesia was given using a subcutaneous injection of buprenorphine (0.1 mg/Kg) in 60 µL of 0.9% saline. A full-thickness circular (10 mm) excisional wound on the back of each mice was created. Subsequently, each mouse wound was inoculated with bioluminescent *S. aureus* (Xen 29) 2.5 × 10^7^ CFU in 10 µL PBS, as previously described [36]. Infected wounds were imaged post-inoculation using Xenogen IVIS Bioluminescent Live Animal Imaging (Caliper Life Sciences, Massachusetts, America) and then daily until sacrifice to quantify radiance and correlate the wound bacterial load. The total photon emission from defined wound areas within the images, region of interest (ROI) was quantified with Living Image R software following established protocols [36]. At two-day post-inoculation, once the bacterial infection was well established, and the presence of mature biofilms was evident by the formation of extracellular polymeric substance (EPS), the wounds were treated daily with either 60 µL of AgNP hydrogel (200 µg/g) equivalent to (12 μg of silver) per treatment, blank hydrogel, and 1% Ag SD cream equivalent to (302 μg of silver) until day 9 and imaged daily to monitor the bacterial burden [24]. Wounds were covered with a sterile Tegaderm^TM^ dressing daily from day 0 to day 10 and post each treatment to create a fertile environment for the development of mature bacterial biofilms and mimic the clinical environment [37]. The infected wounds were also digitally photographed daily throughout the whole trial for macroscopic assessment of wound healing. On the 10th day of the study, all mice were sacrificed, wounds were excised, and major organs (liver, spleen, lung, and kidney) were collected. The wound tissue was halved through the center and half of the tissue was submitted for fixation and subsequent hematoxylin and eosin (H&E) tissue staining for histological analysis of wound healing. The remaining half of the wound was bisected, a quarter was used for bacterial colony counts, and a quarter for live/dead bacterial viability staining [24]. Collected organs were weighed and processed for H&E staining.

### 2.9. Macroscopic and Microscopic Analysis of Wound Healing

Daily digital wound photographs (0–10 day post-surgery) were used for macroscopic assessment of healing using the ImageProPlus program (Media Cybernetics, Inc., Bethesda, MD, America). This included measurements of surface wound area and gape, which were determined by tracing around the wound margin and measuring the distance across the wound bed, respectively, following established protocols [38]. For microscopic analysis of wound healing, we used histology assessments. Collected wound tissue and major organs from each group of mice were fixed in 10% neutral buffered formalin, processed routinely into paraffin, and sectioned at 4 μm thickness using a microtome (Moss Instruments MHS45) [39]. Sections were then processed and stained for hematoxylin and eosin (H&E) using established protocols [40]. Masson trichrome staining was also implemented to assess the total collagen content using previously described methods [41]. The sections were imaged using Olympus IX81 light microscope (Olympus, Tokyo, Japan) and the ImageProPlus program (Media Cybernetics, Inc., Bethesda, MD, America) to microscopically assess the healing by measurements of wound length, wound gape, and percentage of wound re-epithelisation following established methods [24].

### 2.10. In Vivo Antibacterial Assessments

#### 2.10.1. CFU Counts

The number of viable *S. aureus* bacteria from excised wound tissue were determined using the CFU spot plate method in sterile conditions and colony counts were further verified by IVIS bioluminescence imaging of the plates. Tissue samples were resuspended in 200 µL PBS and homogenized by 3× vortexing and 2 min sonication [36]. The homogenates were serially diluted in PBS (20 µL + 180 µL) and spot-plated on the TSA + kanamycin plate allowing for selection of *S. aureus*. The grown visible colonies were manually counted after overnight incubation (18 h) and verified using bioluminescent imaging following established protocols [24].

#### 2.10.2. Bacterial Viability (Live/Dead Staining)

The collected wound tissue was prepared for staining with the Baclight™ viability kit, as described in the previous section [24]. Briefly, tissue samples were washed with PBS and then stained with the live/dead stain and imaged using CLSM following standard protocols as per manufacturers’ recommendations [24].

### 2.11. Statistical Analysis

Results are presented as mean ± standard deviation (SD) unless otherwise stated. The data were analyzed by Student’s *t*-test or one-way ANOVA. When the statistical analysis was significant (*p* < 0.05), post hoc comparisons were conducted using Dunnett’s multiple comparisons. All statistical analysis was performed using GraphPad Prism version 8.0 (GraphPad, Sacramento, California, America). * *p* < 0.05, ** *p* < 0.01, *** *p* < 0.001, and **** *p* < 0.0001.

## 3. Results and Discussion

### 3.1. Preparation and Characterization of AgNP Hydrogel

**Figure 1:**
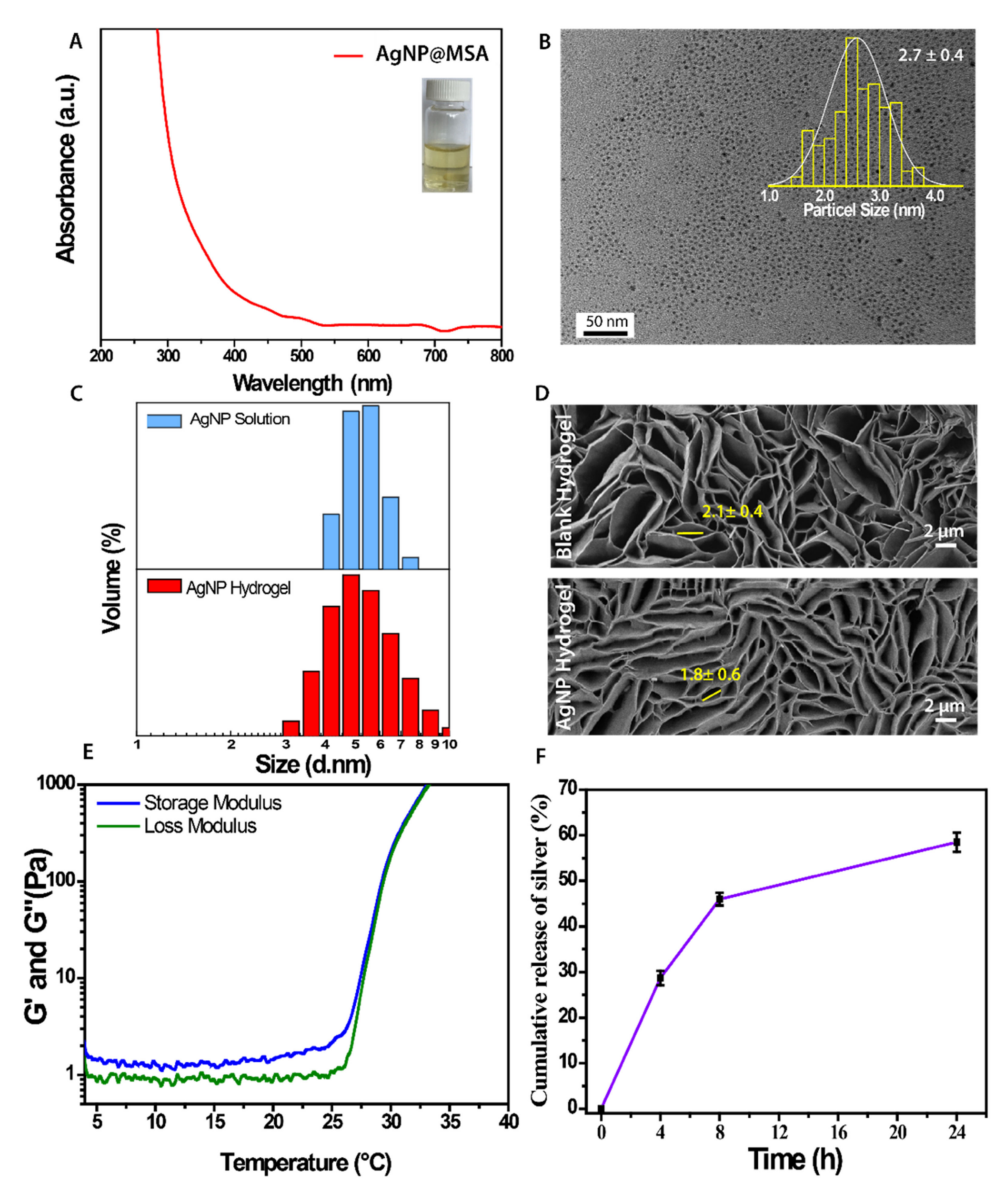
Physicochemical characterizations of AgNP hydrogel. (**A**) UV-vis spectrum of MSA_AgNP with respective solution colour. (**B**) TEM micrographs of AgNPs in water with corresponding size distribution. (**C**) The hydrodynamic diameter of both AgNPs in solution and hydrogel. (**D**) microstructure of both AgNP hydrogel and blank analyzed by SEM. (**E**) AgNP hydrogel gelation point in response to the surrounding temperature. (**F**) In vitro cumulative release of silver from the hydrogel.

Synthesis of ultrasmall silver nanoparticles capped with mercaptosuccinic acid (MSA) was carried out using a protocol previously described [33] and schematically presented in Scheme 1. Here the MSA molecules served as both the primary capping agent and for enhancing the colloidal stability of the AgNPs. The formation of AgNPs was verified using UV vis spectroscopy. As shown in Figure 1A, the absorption peak has no defined prominent peak, suggesting the absence of surface plasmon resonance (SPR). The absence of SPR and the nature of this peak is a strong indication of typical characteristics of ultrasmall AgNPs as we previously demonstrated [32]. The as-prepared AgNPs were light yellow under ambient light (Figure 1A inset). The primary size of AgNPs was verified using TEM displaying a highly monodispersed AgNPs with an average core size of 2.7 nm, hence referred to as ultrasmall AgNPs (<3 nm) (Figure 1B). The incorporation of AgNPs was carried out by adding the pre-made AgNP solution into synthesized pluronic f-127 under continuous stirring to obtain the AgNP loaded hydrogel. Pluronic hydrogels are gaining research interests for drug delivery and tissue engineering applications due to their high biocompatibility, stability with nanoparticles, and thermoresponsive properties. Subsequently, the hydrodynamic diameter of AgNPs was measured when incorporated into the hydrogel. The DLS measurement shows that AgNPs diameter remains unchanged (<5 nm) to those AgNPs in solution (Figure 1C).

The stability of the AgNPs exposed to biological media was also assessed. The result showed that the AgNP hydrogel can maintain its original composition and structure as measured by consistent hydrodynamic diameter after 24 h of exposure to DMEM (10% FBS), Figure S1. Here the collective impact of ultrasmall well-dispersed particles coupled with MSA molecule on the surface of particles acts as an important parameter limiting the immediate formation of protein corona [42]. Additionally, the protein corona formation also depends on the types of protein and incubation period, where longer incubation or circulation could greatly expose the formation of hard or soft corona [43]. The microstructure of both blank and AgNP hydrogel was analysed using Scanning Electron Microscopy (SEM). The SEM images displayed a porous structure with high interconnectivity for both hydrogels with a small change in pore size (Figure 1D). The comparison images show that the blank hydrogel pore is slightly larger (2.1 ± 0.4 µm vs. 1.8 ± 0.6 µm) after AgNP loading however this was not significant. High porosity including the pore size of hydrogel is an important physical property impacting drug diffusion, tissue regeneration, and oxygen and waste removal for cells [44].

The mechanical properties of AgNP loaded and blank hydrogels were measured using a rheometer, showing a similar relationship, irrespective of AgNP addition (Figure S2). The hydrogel presents a typical non-Newtonian fluid behaviour at ambient temperature, where a change in viscosity in response to a change in shear is observed (Figure S2) [45]. The thermoresponsive properties of the AgNP hydrogel were evident through rapid changes in physical properties in response to surrounding temperature (Figure 1E). AgNP hydrogel displayed trends of increasing storage modulus (G’) and loss modulus (G’’) in response to increased temperature with a characteristic cross-over at near-physiological temperature, as indicated where the G’ equilibrate G”, which is defined as the gelation point [46]. This measurement was further supported when the hydrogel viscosity was increased at the defined temperature range (Figure S3). These important viscoelastic properties of the AgNP hydrogel offer ease and flexibility for application, which can be performed using a syringe with an acceptable flow rate, through a conventional topical dispenser or even a laboratory pipette, demonstrating a high degree of hydrogel plasticity (Figure S4). The release of silver from hydrogel was studied over a 24 h period. It displayed a two-phase behaviour release pattern, where the first 6 h showed a fast silver release, followed by a slow and sustained pattern afterward (Figure 1F). This is a consistent pattern for typical sustained release therapeutics [47]. Furthermore, this type of release pattern promotes pathogen elimination, while providing sufficient Ag^+^ concentration to prevent recurrent infections when wounds are undergoing repair mode.

### 3.2. In Vitro Biocompatibility of the AgNP Hydrogel

**Figure 2:**
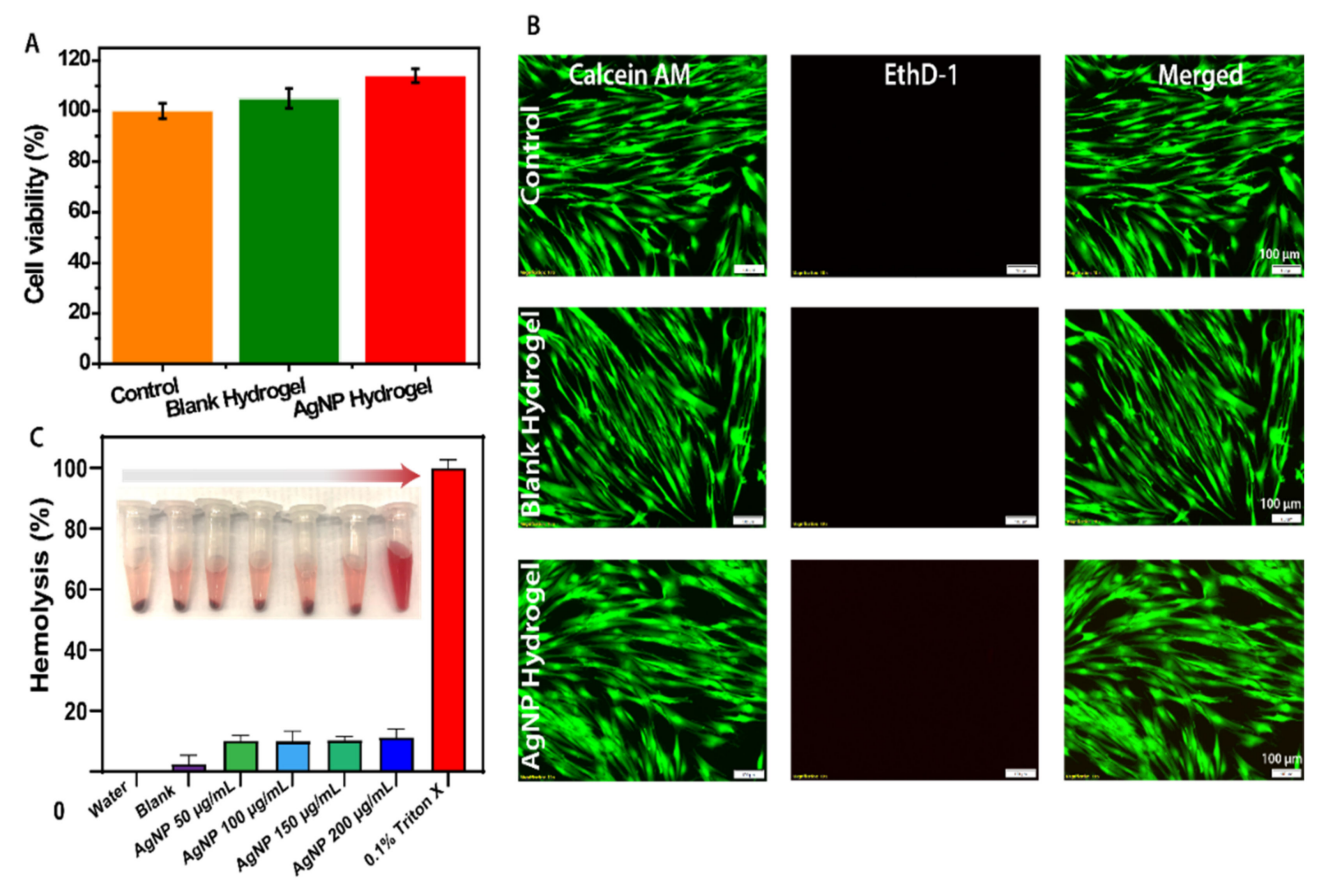
In vitro biocompatibility and hemocompatibility in response to treatment groups. (**A**) HFFs cell viability (%) after 24 h exposure to treatments. (**B**) Fluorescent images of live/dead assay showing cell viability. (**C**) Hemolysis (%) of red blood cells treated with different concentrations of AgNP hydrogel. Data are shown as mean ± SD. Scale bar 100 μm.

Biocompatibility is an essential factor for the safe clinical application of the AgNP hydrogel. The in vitro cytotoxicity of the developed AgNP hydrogel was assessed against human foreskin fibroblasts (HFFs). Fibroblasts were chosen based on their important role in the wound healing process. The cells remain viable when exposed to either blank or AgNP hydrogel after 24 h of exposure, compared to untreated control (Figure 2A,B). As shown in Figure 2A, fibroblast viability was similar and not statistically significant between the treatment groups. A slightly higher cell viability over 100% is often common in metabolic assays due to different proliferation rate and response to the treatment group. Additionally, the live/dead staining was used to semi-qualitatively measure the fibroblast viability, while also observing cell morphology. The HFFs retained maximum viability after 24 h exposure in all groups, as indicated by a higher ratio of green fluorescence intensity with no major change to cell morphology (Figure 2B). The cell viability assay suggests the potential safe application of AgNP hydrogel for applications in wound management. This further supports our previous studies showing the concentration-dependent cell viability of AgNPs against mammalian cells [32].

### 3.3. Hemocompatibility of the AgNP Hydrogel

Hemocompatibility of the AgNP hydrogel was investigated in vitro by measuring the absorbance of released hemoglobin after RBC lysis. AgNP hydrogels with different AgNP concentrations ranging from 50 to 200 μg/mL were set as experimental groups, while water and Triton X-100 were used as negative and positive controls, respectively. The supernatant in all experimental groups displayed a pale pink colour with red blood cell pellet at the bottom of the tube, similar to the water (negative control), while Triton X-100 (positive control) showed a bright red supernatant due to ruptured red blood cells (Figure 2C inset). Furthermore, as shown in Figure 2C and consistent with macroscopic colour observation, the AgNP hydrogel in all tested concentrations (50–200 µg/mL) quantitatively displays lower than 10% hemolysis of RBCs, compared to a positive control (Triton X-100). Indeed, the colour observation and quantitative analysis both support the good hemocompatibility of the AgNP hydrogel comparable to that of water (negative control).

### 3.4. In Vitro Antibiofilm Activity of the AgNP Hydrogel

**Figure 3:**
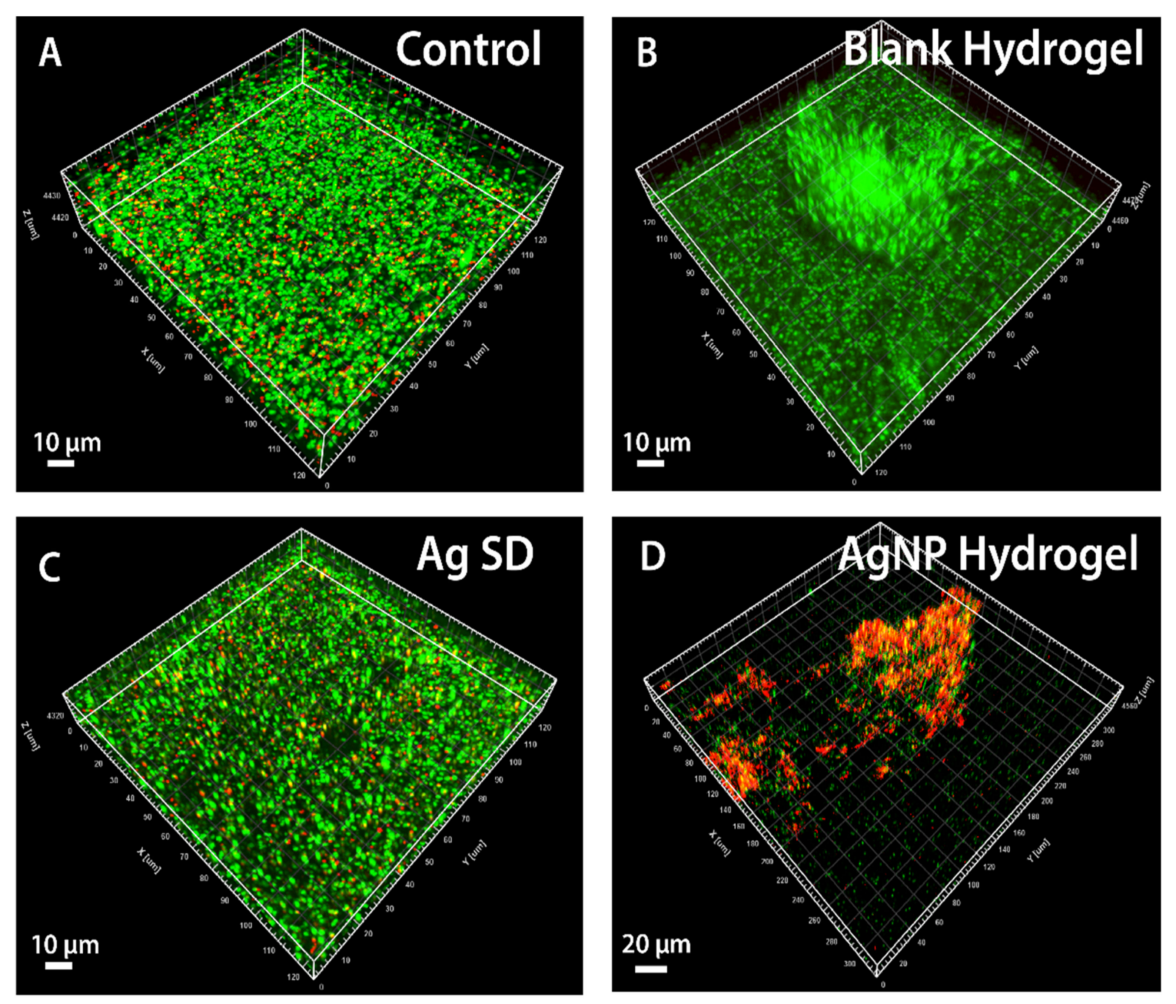
In vitro antibiofilm activity of the AgNP hydrogel. (**A**–**D**) CLSM 3D micrographs of *S. aureus* biofilm structure after treatment with bacterial broth, blank, Ag SD, and AgNP hydrogel.

The in vitro antibiofilm activity of the AgNP hydrogel (200 µg/mL) was carried out for 24 h using *S. aureus* (Xen 29), similar to our previously established protocol [24]. This bacterium was selected for analysis to provide insight into its biofilm formation and susceptibility to AgNPs prior to use in the in vivo mature biofilm wound infection study. Firstly, mature biofilms of *S. aureus* were established and treated with a predetermined concentration of AgNP hydrogel, while Ag SD served as the positive control and saline served as the negative control. As shown in Figure 3A–D, there was a striking difference in biofilm composition, as measured via live/dead staining of the 3D biofilm structure. The SYTO9 exhibited green fluorescence in all live bacteria cells, and a fluorescence emission shift toward red (PI) was observed when the bacterial membrane is damaged, indicating dead bacterial cells. The SYTO9/PI staining of 3D mature *S. aureus* biofilms was mostly stable and intact in structure, as indicated by the high green fluorescence with live cells in response to both control and blank hydrogel treatments (Figure 3A,B). Treatment with current clinical gold standard and positive control Ag SD resulted in physical damage to the cells, as indicated by the increased dead cells while failing to disrupt the structure of mature biofilms. In contrast, treatment with the AgNP hydrogel resulted in a striking difference. Most of the *S. aureus* cells were killed, leading to severe disruption of mature biofilms, as indicated by high red fluorescence intensity fragments with significantly reduced biofilm biomass (Figure 3D). Hence, this observation indicates the strong in vitro potential of the AgNP hydrogel against mature and established *S. aureus* biofilms.

### 3.5. In Vivo Anti-Biofilm Effect of the AgNP Hydrogel 

**Figure 4:**
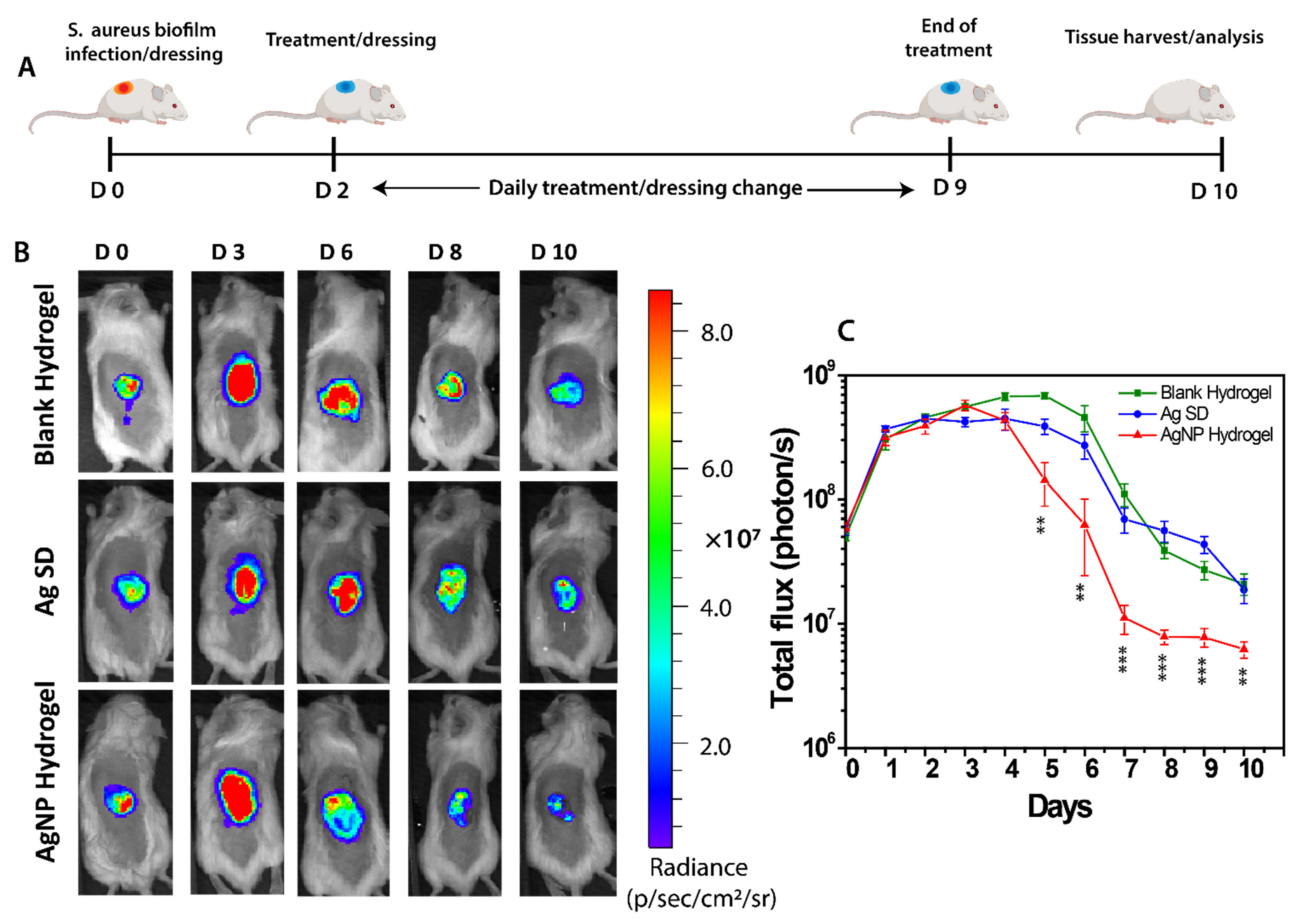
In vivo therapeutic effect of AgNP hydrogel against mature *S. aureus* biofilms. (**A**) Schematic illustration of study progression. (**B**) Representative photographs of IVIS bioluminescent signal differences between treatment groups. (**C**) Average bioluminescent signals corresponding to bacterial burden expressed as the average total flux (photons/s) over 10 days of treatment. Data are shown as mean ± SEM and the * denotes significant difference compared to blank hydrogel using Student’s *t*-test ** *p* < 0.01, and *** *p* < 0.001. *n* = 8.

To demonstrate the in vivo potential of the AgNP hydrogel, an established *S. aureus* (Xen 29) mouse model of mature biofilm wound infection. Wounds were inoculated with *S. aureus* (Xen 29) (5 × 10^7^ CFU) and biofilms allowed to mature and establish by covering the wounds with a transparent Tegaderm dressing, which is known to promote bacterial proliferation, as facilitated by a moist environment, leading to the establishment of dense and stabilized mature biofilms in the first 48 h [48,49]. Following the establishment of mature biofilms as evident by the presence of thick extracellular polymeric substance [50], the daily treatment with AgNP hydrogel commenced on day 2 and was monitored in real-time for 10 days using IVIS bioluminescent imaging (Figure 4A). In this mature biofilm model of wound infection, the bacterial load significantly increases over time, reaching the highest intensity at 3-days post-infection (~3.5 × 10^8^ photons/s) for the Ag SD control or AgNP hydrogel treatment, while the blank hydrogel treated group continued to increase peaking at 5 days post-infection (~7.0 × 10^8^ photons/s) (Figure 4B,C). The difference in treatment efficacy was apparent from day 4 onwards, where the AgNP hydrogel treatment gradually reduced the *S. aureus* bacterial load, as quantitively presented in Figure 4C. The treatment with Ag SD was less effective in clearing the infection with only moderate efficacy observed, compared to blank hydrogel at 5 days post-infection. Additionally, antimicrobial effects of Ag SD were not sustained in subsequent days, indicating that Ag SD may require a higher dose (which may also cause cytotoxicity) to combat mature *S. aureus* biofilms or a longer period of continuous treatment to harness full antibacterial activity. In contrast, treatment with AgNP hydrogel at a much lower concentration resulted in efficient *S. aureus* bacterial clearance, which was statistically significant from 5 to 10 days post-infection, illustrating superior efficacy in clearing established mature *S. aureus* biofilms in wounds in vivo (Figure 4A–C).

The endpoint bacterial counts were verified using standard plate count, confirming the strong antimicrobial properties of developed AgNP hydrogel (Figure S5). These data indicate that wound biofilm exposed to daily AgNP hydrogel treatment results in a weakened and disintegrated biofilm structure and highlights the benefits of ultrasmall particles actively penetrating mature biofilms. Effective removal of biofilm depends on the diffusion capacity of antimicrobial agents. A similar observation was also previously reported, showing enhanced penetration of small particles through bacterial biofilms [51]. However, this observation was based on using pyomyositis mice model where the infected muscle was treated with AgNPs injected intramuscularly, hence resulting in higher accumulation and subsequent antibiofilm effect [51]. The successful treatment of biofilms requires the use of optimized AgNPs to facilitate rapid bacterial clearance to prevent systemic infection. Current relevant studies of AgNPs treatments against in vivo skin wound biofilm models are limited to the use of larger AgNP with uncontrolled release mechanisms, and often encounter various stability issues that compromise their potency against bacteria [52,53].

### 3.6. End-Point Tissue Bacterial Biofilm Analysis

**Figure 5:**
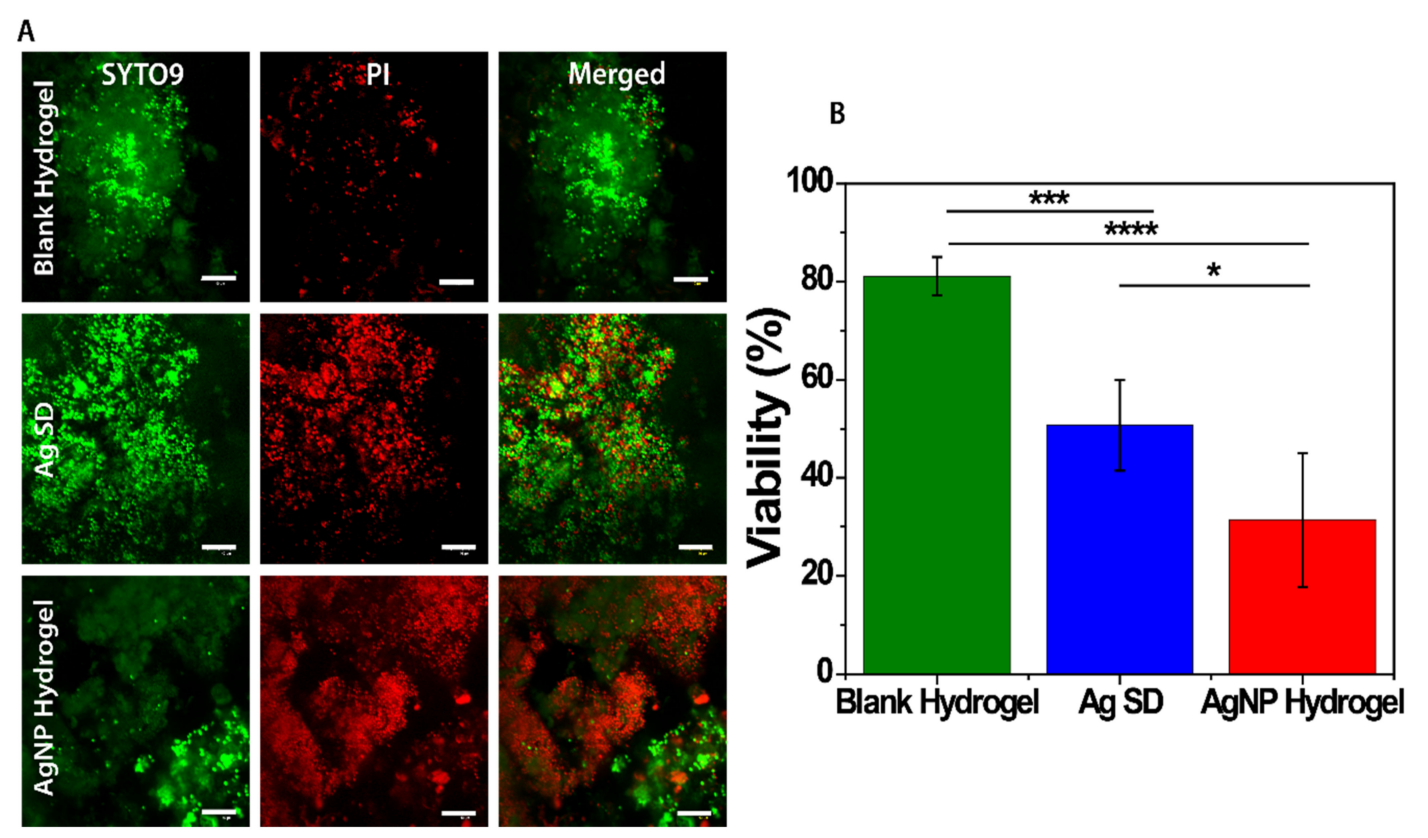
In vivo bacterial tissue biofilm staining and viability. (**A**) CLSM images of *S. aureus* bacteria showing the ratio of SYTO/PI in response to treatment with blank, Ag SD, and AgNP hydrogel treatments. (**B**) The corresponding cell viability of each treatment group. Data are shown as mean ± SD and the * denotes significant difference using one-way ANOVA followed by Dunnett’s multiple comparison test. * *p* < 0.05, *** *p* < 0.001 and **** *p* < 0.0001, Scale bar 10 μm, *n* = 8.

We extended the in vivo biofilm observation by analyzing residual *S. aureus* bacteria within dissected wounds using live/dead staining. Samples were stained with two nucleic acid dyes and the fluorescence intensity of SYTO9/PI was monitored using CLSM. Treatment of infected wounds with blank hydrogel control resulted in high levels of green fluorescence (merged), indicating the presence of a viable mature biofilm and survival of bacteria in response to blank hydrogel (Figure 5A). Treatment with Ag SD positive control resulted in reduced cell viability, with treatment efficacy showing some success with ~50% of *S. aureus* bacteria remaining viable in mature biofilms within the wounds (Figure 5A,B). However, treatment with the AgNP hydrogel showed a marked difference in fluorescence intensity with predominately stained red (dead cells) and more dispersed *S. aureus* biofilms within the wounds. These observations were supported with quantitative analysis showing that the AgNP hydrogel treatment resulted in only ~30% bacterial viability within wound biofilms while the Ag SD and blank hydrogel treatments resulted in 50% and 80% viability, respectively (Figure 5B). The increased membrane permeability (red fluorescence) in response to AgNP hydrogel treatment further supports the role of AgNPs in targeting mature biofilms and leading to improved bacterial killing efficiency. Unlike larger particles or silver salts (Ag SD), the small-sized AgNPs have a significantly higher affinity and tendency to interact with the complex structures of mature biofilms to target bacterial cells [54]. These observations are consistent with our previous study, suggesting that ultra-small AgNPs can effectively damage the structural integrity of the *S. aureus* cell membrane and increase permeability, resulting in a higher degree of bacterial eradication [33].

### 3.7. In Vivo Assessment of AgNP Hydrogel Efficacy in Promoting Healing of Infected Wounds

**Figure 6:**
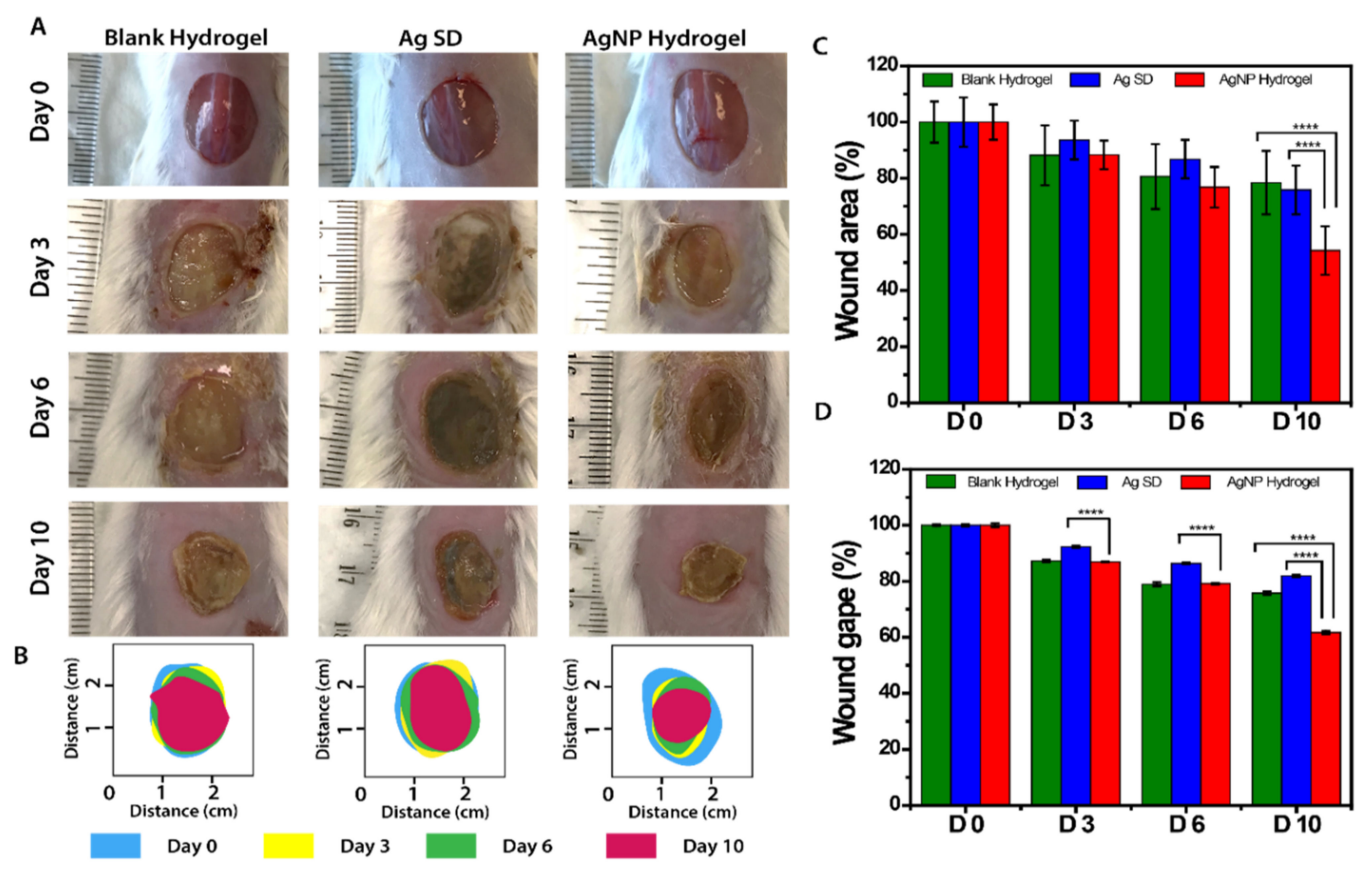
Macroscopic analysis of *S. aureus* infected wound healing following AgNP treatment. (**A**,**B**) digital photographs and schematic tracing of wound closure in response to treatment. (**C**,**D**) quantitative analysis of wound area (%) and wound gape (%) at different time points. Data are shown as mean ± SD and the * denotes significant difference using one-way ANOVA followed by Dunnett’s multiple comparison test. **** *p* < 0.0001, *n* = 8.

The effect of AgNP hydrogel on healing of *S. aureus* infected wounds was assessed using macroscopic analysis of surface wound area and gape using digital photographs. Quantitative analysis showed that both the blank hydrogel and Ag SD treatments resulted in slower healing with only ~20% of surface wound closure compared to day 0 over 10 days in both groups, as illustrated by measurements of wound area and gape (Figure 6A–D). In contrast, the AgNP hydrogel treatment resulted in a significantly faster wound closure rate, with ~45% of surface wound closure compared to day 0 over the 10 days period and a concurrent decrease in both wound area and gape, compared to both blank hydrogel and Ag SD treatment controls, which was most pronounced on day 10 of the study (Figure 6A–D).

**Figure 7:**
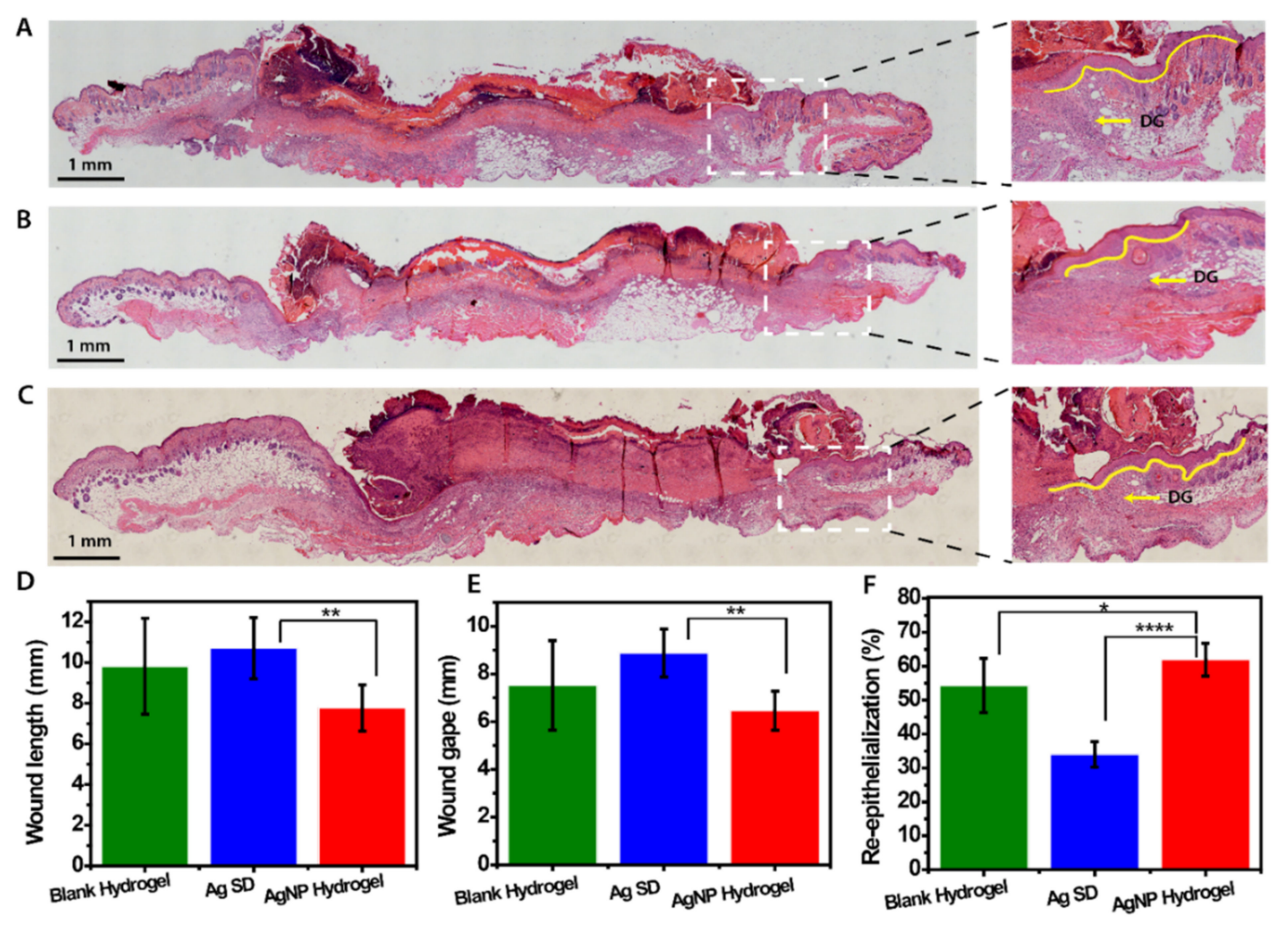
AgNP treatment improves healing and wound reepithelization. H&E-stained blank hydrogel (**A**), Ag SD (**B**), and AgNP hydrogel (**C**) treated wounds with enlarged areas illustrating dermal gape (yellow arrow) and wound re-epithelization (yellow line). Quantitative analysis of wound length (**D**), wound gape (**E**), and percent of wound re-epithelization (**F**) at day 10 post wounding. Data are shown as mean ± SD and the * denotes significant difference using one-way ANOVA followed by Dunnett’s multiple comparison test. * *p* < 0.05, ** *p* < 0.01, and **** *p* < 0.0001, *n* = 8.

To further elucidate the capacity of the AgNP hydrogel to promote healing of *S. aureus* infected wounds, histological analysis of the wounds at the 10-day endpoint was performed, including analysis of wound length, wound gape, and wound re-epithelialization. Treatment of infected wounds with the AgNP hydrogel resulted in significantly smaller wound areas and dermal wound gapes, compared to Ag SD treatment, which showed a trend towards delaying healing compared to blank hydrogel treatment, where only a minimal improvement in wound healing was observed (Figure 7A–F). A striking finding was the significant improvement in wound re-epithelialization observed with AgNP treatment. *S. aureus* infected wounds treated with AgNP hydrogel showed a 62% improvement in wound reepithelization compared to Ag SD (30%), which is considered the current gold standard treatment, and blank hydrogel control (53%) (Figure 7F). Clinical management of infected chronic wounds has been challenging and number of different combinatory approaches are often employed to achieve wound regeneration. In particular, use of autologous platelet rich plasma (PRP) and cellular therapies, including stromal vascular fraction cells (SVFs) and adipose stem cells (ASCs), are commonly used as treatments for tissue regeneration [55]. Incorporation of AgNP in these therapy approaches, or tissue engineered scaffolds, offers an opportunity for added antimicrobial functionality to prevent bacterial growth and minimize infection. Application of ASCs to wounds has been shown to promote healing at different stages of wound repair by reducing inflammation and promoting cellular proliferation [56] and studies have shown that large AgNP do now cause cytotoxicity to undifferentiated ASCs and can stimulate ASCs secretion of cytokines that promote tissue regeneration [57], suggesting benefits of a combined treatment approach. In the context of the presented results, a previous clinical study has reported that combined PRP and biofunctionalized hyaluronic acid requires 30 days of treatment to achieve 96.7% reepithelialization in human treated samples, compared to our study showing that AgNP hydrogel can improve reepithelization by 62% within 10 days of treatment of the infected mouse wound model [58]. This suggests that a combined treatment with PRP therapy and AgNP may potentially offer more desirable patient outcomes in terms of both management of wound infection and promotion of tissue regeneration and this should be further explored.

**Figure 8:**
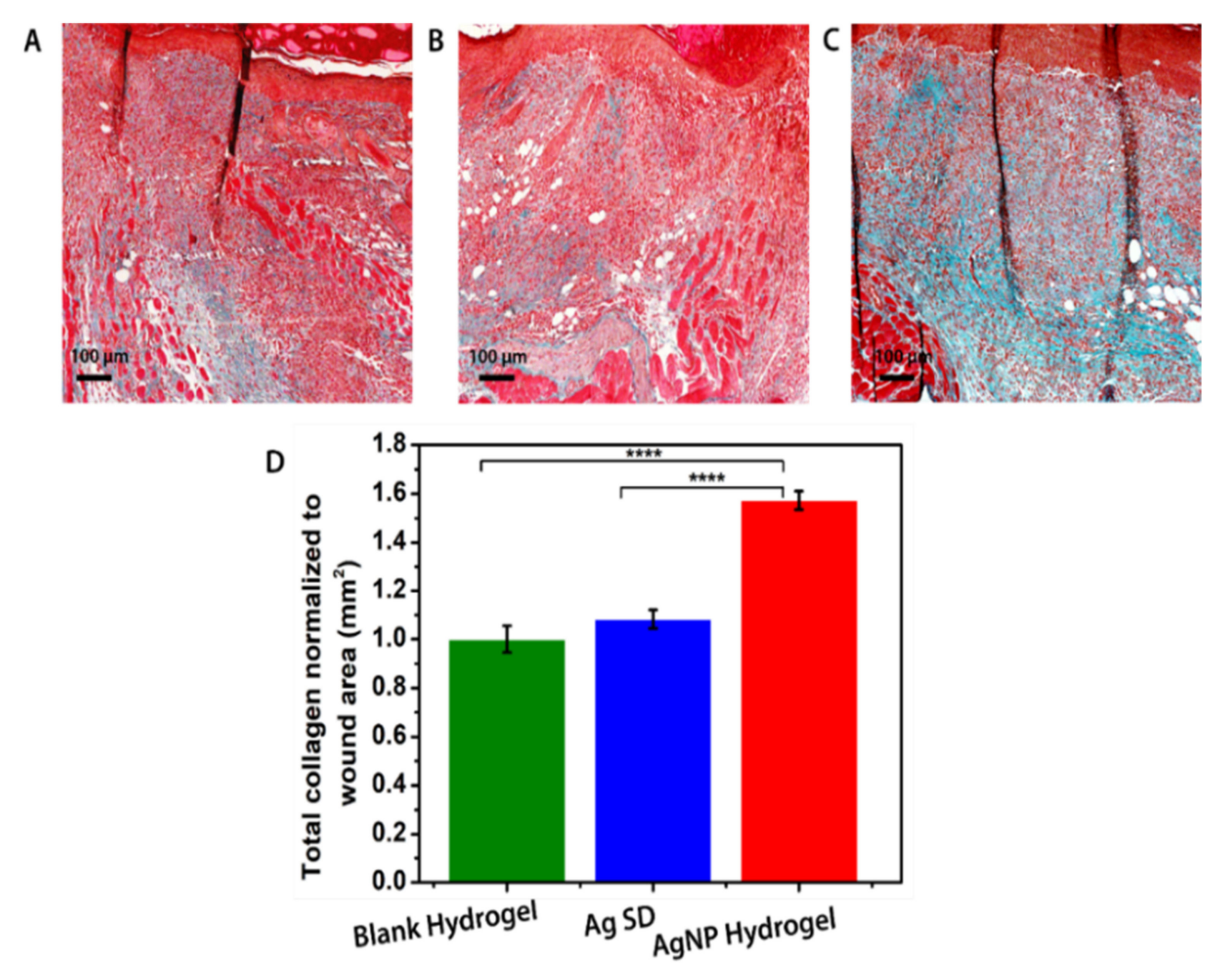
Masson’s trichrome staining of the wound sections showing collagen deposition after treatment for 10 days. (**A**–**C**) Images of Masson’s trichrome stained wound tissues of blank hydrogel, Ag SD, and AgNP hydrogel displaying collagen distribution and arrangements. (**D**) Quantitative analysis of total collagen content detected normalized to the respective wound area (mm^2^). Data are shown as mean ± SD and the * denotes significant difference using one-way ANOVA followed by Dunnett’s multiple comparison test. **** *p* < 0.0001, *n* = 8.

Considering observed improvements in the rate of healing and re-epithelization following AgNP hydrogel treatment, we next investigated the impact of AgNP hydrogel on the early deposition of total collagen within the wound bed. Early collagen deposition in the wound is a good predictive indicator of the quality of healing as well as indicating potential impacts on downstream outcomes of tissue regeneration and scarring. Masson’s Trichrome staining for total collagen showed a more dense and organized deposition of collagen in the wound beds of AgNP hydrogel treated mice compared to Ag SD and blank hydrogel treated controls (Figure 8A–C). Quantitative analysis of collagen content within the wound bed showed significantly higher levels of total collagen within the wound bed in response to AgNP hydrogel treatment compared to both Ag SD and blank hydrogel control groups (Figure 8D).

Taken together, these results demonstrate that AgNP hydrogel promotes wound healing, which is consistent with a previous study using a planktonic bacteria wound model, which also showed an improved rate of wound closure in response to AgNP hydrogel treatment [59]. These results could be attributed to the combined effect of hydrogel biocompatibility and cell proliferative and antibacterial properties of ultra-small AgNPs to effectively eliminate bacterial pathogens and allow wound regeneration. Other studies have also emphasized the importance of rapid bacterial clearance from the wound to allow wound recovery post-infections [5,60]. Healing-promoting properties of the AgNP hydrogel can be attributed to the biocompatibility of the hydrogel, sustained release of Ag^+^ to clear infections, and wound healing functionalities of the pluronic hydrogel. Recently, a number of treatment modalities including both cellular therapies and biofunctionalized materials have gained momentum in regenerative medicine; however, treatment outcomes have been variable and often unsatisfactory as wound infection is still a critical challenge [61]. It is becoming increasingly clear that healing and regeneration of chronic wounds requires powerful and robust antimicrobial agents to clear infection and provide protection against recurrent infections through sustained and prolonged antimicrobial activity [62]. Here we have shown that our AgNP hydrogel not only clears infections by eradicating wound biofilms but also significantly improves the regenerative process through its multifunctional properties. Future studies should examine the effect of this AgNP delivery system on wound regeneration and complete healing in combination with some of the advances wound therapies currently in use, including PRP and ASCs, in order to direct development of next generation of wound-healing therapies.

### 3.8. Safety of the AgNP Hydrogel Treatment

**Figure 9:**
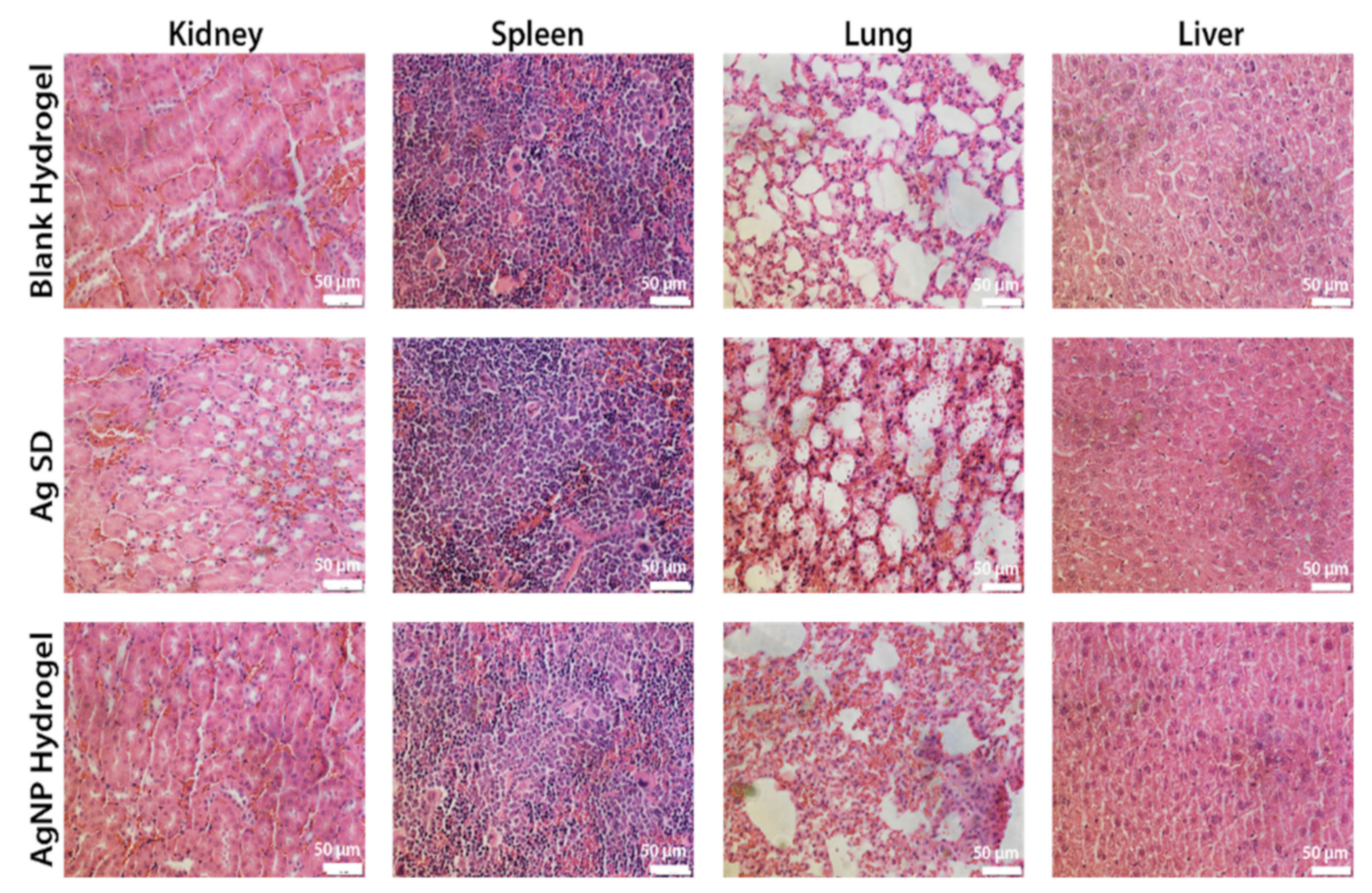
Hematoxylin-eosin (H&E) staining of the major organs after 10 days of different treatments, Scale bar 50 μm, *n* = 8.

To address the concerns associated with silver toxicity, the biosafety of AgNP hydrogel treatment was investigated using histological analysis of major organs, including kidney, spleen, lung, and liver. No detectable signs of organ toxicity, silver deposition, development of inflammatory lesions, or any pathological structural changes caused by deposition of metal particles were observed after 10 days of treatment in all tested groups (Figure 9). These results further support the safe topical application of the AgNP hydrogel as a novel antimicrobial treatment for *S. aureus* infected wounds, highlighting the developed AgNP hydrogel as an attractive therapeutic option for the treatment of any kind of antibiotic-resistant infected chronic wounds.

## 4. Conclusions

In summary, this study has proposed and validated the use of an AgNP hydrogel for effective and safe elimination of mature *S. aureus* biofilms, facilitating infection clearance and promotion of wound healing in vivo. The AgNP hydrogel was able to demonstrate sufficient AgNP release capacity while also being able to maintain original delicate properties to facilitate AgNP accumulation and penetration of mature *S. aureus* biofilms, resulting in biofilm disintegration through the coordinated size-related pathways. The in-vivo studies using the mature biofilm wound infection model demonstrated the strong capacity of the developed AgNP hydrogel to eliminate infection while extending the inherent benefits of accelerated wound closure and re-epithelization coupled with improved deposition of collagen within the wound bed. Importantly, this multifunctional hydrogel delivery system not only controls *S. aureus* infection but also accelerates the healing of infected wounds, hence providing a safe and promising therapeutic approach in clinical wound care where biofilm-associated infections impair the healing of chronic wounds.

## Data Availability

Data available on request from co-authors.

## Supplementary Materials

The following are available online at https://www.mdpi.com/article/10.3390/biomedicines9091182/s1, Table S1: Pluronic F-127 hydrogel formulation composition, Figure S1: Dynamic light scattering (DLS) size distribution of AgNP hydrogel is measured as volume (%). (a,b) Mean hydrodynamic diameter of AgNP hydrogel in DMEM (10% FCS) at t = 0. and t = 24 at 37 °C storage, Figure S2: Measurement of shear stress in response to shear rate of pluronic f-127 hydrogel at ambient temperature, Figure S3: Measurement of shear stress in response to shear rate of pluronic f-127 hydrogel at ambient temperature, Figure S4: Photograph of AgNP hydrogel delivered using a syringe demonstrating the ease of application and excellent hydrogel viscoelastic properties, Figure S5: CFU counts of residual bacteria within wound tissue after 10 days of treatment. Data are shown as mean ± SD and the * denotes significant difference using one-way ANOVA followed by Dunnett’s multiple comparison test. * *p* < 0.05, *n* = 8.

## References

[1] Koo H., Allan R.N., Howlin R.P., Stoodley P., Hall-Stoodley L. (2017). Targeting microbial biofilms: Current and prospective therapeutic strategies. Nat. Rev. Microbiol..

[2] Frieri M., Kumar K., Boutin A. (2017). Antibiotic resistance. J. Infect. Public Health.

[3] Flemming H.-C., Wingender J., Szewzyk U., Steinberg P., Rice S.A., Kjelleberg S. (2016). Biofilms: An emergent form of bacterial life. Nat. Rev. Microbiol..

[4] Kostakioti M., Hadjifrangiskou M., Hultgren S.J. (2013). Bacterial biofilms: Development, dispersal, and therapeutic strategies in the dawn of the postantibiotic era. Cold Spring Harb. Perspect. Med..

[5] Liu W., Ou-Yang W., Zhang C., Wang Q., Pan X., Huang P., Zhang C., Li Y., Kong D., Wang W. (2020). Synthetic Polymeric Antibacterial Hydrogel for Methicillin-Resistant *Staphylococcus aureus*-Infected Wound Healing: Nanoantimicrobial Self-Assembly, Drug- and Cytokine-Free Strategy. ACS Nano.

[6] Gao Y., Wang J., Chai M., Li X., Deng Y., Jin Q., Ji J. (2020). Size and Charge Adaptive Clustered Nanoparticles Targeting the Biofilm Microenvironment for Chronic Lung Infection Management. ACS Nano.

[7] Han G., Ceilley R. (2017). Chronic Wound Healing: A Review of Current Management and Treatments. Adv. Ther..

[8] Mi G., Shi D., Wang M., Webster T.J. (2018). Reducing Bacterial Infections and Biofilm Formation Using Nanoparticles and Nanostructured Antibacterial Surfaces. Adv. Funct. Mater..

[9] Haidari H., Garg S., Vasilev K., Kopecki Z., Cowin A.J. (2020). Silver-based wound dressings: Current issues and future developments for treating bacterial infections. Wound Pract. Res..

[10] Liu Y., Shi L., Su L., van der Mei H.C., Jutte P.C., Ren Y., Busscher H.J. (2019). Nanotechnology-based antimicrobials and delivery systems for biofilm-infection control. Chem. Soc. Rev..

[11] Cheng H., Li Y., Huo K., Gao B., Xiong W. (2014). Long-lasting in vivo and in vitro antibacterial ability of nanostructured titania coating incorporated with silver nanoparticles. J. Biomed. Mater. Res. Part A.

[12] Kalantari K., Mostafavi E., Afifi A.M., Izadiyan Z., Jahangirian H., Rafiee-Moghaddam R., Webster T.J. (2020). Wound dressings functionalized with silver nanoparticles: Promises and pitfalls. Nanoscale.

[13] Barbalinardo M., Antosova A., Gambucci M., Bednarikova Z., Albonetti C., Valle F., Sassi P., Latterini L., Gazova Z., Bystrenova E. (2020). Effect of metallic nanoparticles on amyloid fibrils and their influence to neural cell toxicity. Nano Res..

[14] Decataldo F., Barbalinardo M., Gentili D., Tessarolo M., Calienni M., Cavallini M., Fraboni B. (2020). Organic Electrochemical Transistors for Real-Time Monitoring of In Vitro Silver Nanoparticle Toxicity. Adv. Biosys..

[15] Rao B.R., Kotcherlakota R., Nethi S.K., Puvvada N., Sreedhar B., Chaudhuri A., Patra C.R. (2018). Ag_2_[Fe(CN)_5_NO] Nanoparticles Exhibit Antibacterial Activity and Wound Healing Properties. ACS Biomater. Sci. Eng..

[16] Baek K., Liang J., Lim W.T., Zhao H., Kim D.H., Kong H. (2015). In Situ Assembly of Antifouling/Bacterial Silver Nanoparticle-Hydrogel Composites with Controlled Particle Release and Matrix Softening. ACS Appl. Mater. Interfaces.

[17] Dai T., Wang C., Wang Y., Xu W., Hu J., Cheng Y. (2018). A Nanocomposite Hydrogel with Potent and Broad-Spectrum Antibacterial Activity. ACS Appl. Mater. Interfaces.

[18] Urzedo A.L., Gonçalves M.C., Nascimento M.H.M., Lombello C.B., Nakazato G., Seabra A.B. (2020). Cytotoxicity and Antibacterial Activity of Alginate Hydrogel Containing Nitric Oxide Donor and Silver Nanoparticles for Topical Applications. ACS Biomater. Sci. Eng..

[19] Taheri S., Cavallaro A., Christo S.N., Smith L.E., Majewski P., Barton M., Hayball J.D., Vasilev K. (2014). Substrate independent silver nanoparticle based antibacterial coatings. Biomaterials.

[20] Haidari H., Kopecki Z., Sutton A.T., Garg S., Cowin A.J., Vasilev K. (2021). pH-Responsive “Smart” Hydrogel for Controlled Delivery of Silver Nanoparticles to Infected Wounds. Antibiotics.

[21] GhavamiNejad A., Rajan Unnithan A., Ramachandra Kurup Sasikala A., Samarikhalaj M., Thomas R.G., Jeong Y.Y., Nasseri S., Murugesan P., Wu D., Hee Park C. (2015). Mussel-Inspired Electrospun Nanofibers Functionalized with Size-Controlled Silver Nanoparticles for Wound Dressing Application. ACS Appl. Mater. Interfaces.

[22] Liu J., Li S., Fang Y., Zhu Z. (2019). Boosting antibacterial activity with mesoporous silica nanoparticles supported silver nanoclusters. J. Colloid Interface Sci..

[23] Zhang Y., Sun P., Zhang L., Wang Z., Wang F., Dong K., Liu Z., Ren J., Qu X. (2019). Silver-Infused Porphyrinic Metal–Organic Framework: Surface-Adaptive, On-Demand Nanoplatform for Synergistic Bacteria Killing and Wound Disinfection. Adv. Funct. Mater..

[24] Haidari H., Bright R., Strudwick X.L., Garg S., Vasilev K., Cowin A.J., Kopecki Z. (2021). Multifunctional ultrasmall AgNP hydrogel accelerates healing of *S. aureus* infected wounds. Acta Biomater..

[25] Gurtner G.C., Werner S., Barrandon Y., Longaker M.T. (2008). Wound repair and regeneration. Nature.

[26] Parani M., Lokhande G., Singh A., Gaharwar A.K. (2016). Engineered Nanomaterials for Infection Control and Healing Acute and Chronic Wounds. ACS Appl. Mater. Interfaces.

[27] AshaRani P.V., Low Kah Mun G., Hande M.P., Valiyaveettil S. (2009). Cytotoxicity and Genotoxicity of Silver Nanoparticles in Human Cells. ACS Nano.

[28] Zanette C., Pelin M., Crosera M., Adami G., Bovenzi M., Larese F.F., Florio C. (2011). Silver nanoparticles exert a long-lasting antiproliferative effect on human keratinocyte HaCaT cell line. Toxicol. In Vitro.

[29] Jose Ruben M., Jose Luis E., Alejandra C., Katherine H., Juan B.K., Jose Tapia R., Miguel Jose Y. (2005). The bactericidal effect of silver nanoparticles. Nanotechnology.

[30] Chen G., Yu Y., Wu X., Wang G., Ren J., Zhao Y. (2018). Bioinspired Multifunctional Hybrid Hydrogel Promotes Wound Healing. Adv. Funct. Mater..

[31] Haidari H., Zhang Q., Melville E., Kopecki Z., Song Y., Cowin A.J., Garg S. (2017). Development of Topical Delivery Systems for Flightless Neutralizing Antibody. J. Pharm. Sci..

[32] Haidari H., Goswami N., Bright R., Kopecki Z., Cowin A.J., Garg S., Vasilev K. (2019). The interplay between size and valence state on the antibacterial activity of sub-10 nm silver nanoparticles. Nanoscale Adv..

[33] Haidari H., Kopecki Z., Bright R., Cowin A.J., Garg S., Goswami N., Vasilev K. (2020). Ultrasmall AgNP-Impregnated Biocompatible Hydrogel with Highly Effective Biofilm Elimination Properties. ACS Appl. Mater. Interfaces.

[34] Shakya S., He Y., Ren X., Guo T., Maharjan A., Luo T., Wang T., Dhakhwa R., Regmi B., Li H. (2019). Ultrafine Silver Nanoparticles Embedded in Cyclodextrin Metal-Organic Frameworks with GRGDS Functionalization to Promote Antibacterial and Wound Healing Application. Small.

[35] Liang Y., Zhao X., Hu T., Han Y., Guo B. (2019). Mussel-inspired, antibacterial, conductive, antioxidant, injectable composite hydrogel wound dressing to promote the regeneration of infected skin. J. Colloid Interface Sci..

[36] Ogunniyi A.D., Kopecki Z., Hickey E.E., Khazandi M., Peel E., Belov K., Boileau A., Garg S., Venter H., Chan W.Y. (2018). Bioluminescent murine models of bacterial sepsis and scald wound infections for antimicrobial efficacy testing. PLoS ONE.

[37] Gurjala A.N., Geringer M.R., Seth A.K., Hong S.J., Smeltzer M.S., Galiano R.D., Leung K.P., Mustoe T.A. (2011). Development of a novel, highly quantitative in vivo model for the study of biofilm-impaired cutaneous wound healing. Wound Repair Regen..

[38] Cowin A., Adams D., Strudwick X., Chan H., Hooper J., Sander G., Rayner T., Matthaei K., Powell B., Campbell H. (2007). Flightless I deficiency enhances wound repair by increasing cell migration and proliferation. J. Pathol..

[39] Yu Y., Li P., Zhu C., Ning N., Zhang S., Vancso G.J. (2019). Multifunctional and Recyclable Photothermally Responsive Cryogels as Efficient Platforms for Wound Healing. Adv. Funct. Mater..

[40] Kopecki Z., Arkell R., Powell B.C., Cowin A.J. (2009). Flightless I Regulates Hemidesmosome Formation and Integrin-Mediated Cellular Adhesion and Migration during Wound Repair. J. Investig. Dermatol..

[41] Han L., Lu X., Liu K., Wang K., Fang L., Weng L.-T., Zhang H., Tang Y., Ren F., Zhao C. (2017). Mussel-Inspired Adhesive and Tough Hydrogel Based on Nanoclay Confined Dopamine Polymerization. ACS Nano.

[42] Tenzer S., Docter D., Rosfa S., Wlodarski A., Kuharev J., Rekik A., Knauer S.K., Bantz C., Nawroth T., Bier C. (2011). Nanoparticle Size Is a Critical Physicochemical Determinant of the Human Blood Plasma Corona: A Comprehensive Quantitative Proteomic Analysis. ACS Nano.

[43] Visalakshan R.M., MacGregor M.N., Sasidharan S., Ghazaryan A., Mierczynska-Vasilev A.M., Morsbach S., Mailänder V., Landfester K., Hayball J.D., Vasilev K. (2019). Biomaterial Surface Hydrophobicity-Mediated Serum Protein Adsorption and Immune Responses. ACS Appl. Mater. Interfaces.

[44] Song X., Zhu C., Fan D., Mi Y., Li X., Fu R.Z., Duan Z., Wang Y., Feng R.R. (2017). A Novel Human-Like Collagen Hydrogel Scaffold with Porous Structure and Sponge-Like Properties. Polymers.

[45] Lopez Hernandez H., Grosskopf A.K., Stapleton L.M., Agmon G., Appel E.A. (2019). Non-Newtonian Polymer–Nanoparticle Hydrogels Enhance Cell Viability during Injection. Macromol. Biosci..

[46] Sun Han Chang R., Lee J.C.-W., Pedron S., Harley B.A.C., Rogers S.A. (2019). Rheological Analysis of the Gelation Kinetics of an Enzyme Cross-linked PEG Hydrogel. Biomacromolecules.

[47] Li J., Mooney D.J. (2016). Designing hydrogels for controlled drug delivery. Nat. Rev. Mater..

[48] Feng Y., Wang Q., He M., Zhang X., Liu X., Zhao C. (2019). Antibiofouling Zwitterionic Gradational Membranes with Moisture Retention Capability and Sustained Antimicrobial Property for Chronic Wound Infection and Skin Regeneration. Biomacromolecules.

[49] Kopecki Z., Ogunniyi A.D., Trott D.J., Cowin A. (2017). Fighting chronic wound infection—One model at a time. Wound Pract. Res..

[50] Phillips P.L., Wolcott R.D., Fletcher J., Schultz G.S. (2010). Biofilms Made Easy. Wounds Int..

[51] Wu J., Li F., Hu X., Lu J., Sun X., Gao J., Ling D. (2019). Responsive Assembly of Silver Nanoclusters with a Biofilm Locally Amplified Bactericidal Effect to Enhance Treatments against Multi-Drug-Resistant Bacterial Infections. ACS Cent. Sci..

[52] Le Ouay B., Stellacci F. (2015). Antibacterial activity of silver nanoparticles: A surface science insight. Nano Today.

[53] Patel K.K., Surekha D.B., Tripathi M., Anjum M.M., Muthu M.S., Tilak R., Agrawal A.K., Singh S. (2019). Antibiofilm Potential of Silver Sulfadiazine-Loaded Nanoparticle Formulations: A Study on the Effect of DNase-I on Microbial Biofilm and Wound Healing Activity. Mol. Pharm..

[54] Ferry J.L., Craig P., Hexel C., Sisco P., Frey R., Pennington P.L., Fulton M.H., Scott I.G., Decho A.W., Kashiwada S. (2009). Transfer of gold nanoparticles from the water column to the estuarine food web. Nat. Nanotechnol..

[55] Gentile P., Sterodimas A., Pizzicannella J., Dionisi L., De Fazio D., Calabrese C., Garcovich S. (2020). Systematic Review: Allogenic Use of Stromal Vascular Fraction (SVF) and Decellularized Extracellular Matrices (ECM) as Advanced Therapy Medicinal Products (ATMP) in Tissue Regeneration. Int. J. Mol. Sci..

[56] Gentile P., Garcovich S. (2019). Concise Review: Adipose-Derived Stem Cells (ASCs) and Adipocyte-Secreted Exosomal microRNA (A-SE-miR) Modulate Cancer Growth and promote Wound Repair. J. Clin. Med..

[57] Abdal Dayem A., Lee S.B., Cho S.G. (2018). The Impact of Metallic Nanoparticles on Stem Cell Proliferation and Differentiation. Nanomaterials.

[58] De Angelis B., D’Autilio M.F.L.M., Orlandi F., Pepe G., Garcovich S., Scioli M.G., Orlandi F., Cervelli V., Gentile P. (2019). Wound Healing: In Vitro and In Vivo Evaluation of a Bio-Functionalized Scaffold Based on Hyaluronic Acid and Platelet-Rich Plasma in Chronic Ulcers. J. Clin. Med..

[59] Zhang H., Sun X., Wang J., Zhang Y., Dong M., Bu T., Li L., Liu Y., Wang L. (2021). Multifunctional Injectable Hydrogel Dressings for Effectively Accelerating Wound Healing: Enhancing Biomineralization Strategy. Adv. Funct. Mater..

[60] Huang W.-C., Ying R., Wang W., Guo Y., He Y., Mo X., Xue C., Mao X. (2020). A Macroporous Hydrogel Dressing with Enhanced Antibacterial and Anti-Inflammatory Capabilities for Accelerated Wound Healing. Adv. Funct. Mater..

[61] Gentile P., Garcovich S. (2021). Systematic Review: Adipose-Derived Mesenchymal Stem Cells, Platelet-Rich Plasma and Biomaterials as New Regenerative Strategies in Chronic Skin Wounds and Soft Tissue Defects. Int. J. Mol. Sci..

[62] Kopecki Z. (2021). Development of next-generation antimicrobial hydrogel dressing to combat burn wound infection. Biosci Rep..

