# Supplementary material for: Eradication of Mature Bacterial Biofilms with Concurrent Improvement in Chronic Wound Healing Using Silver Nanoparticle Hydrogel Treatment
Source: Biomedicines. 2021 Sep 8;9(9):1182. doi: 10.3390/biomedicines9091182 (PMC8470956; doi:10.3390/biomedicines9091182)
Supplement: Supplementary file 1 [file biomedicines-09-01182-s001.zip › biomedicines-1365724-supplementary.pdf]

Supplementary Materials

Eradication of Mature Bacterial Biofilms with Concurrent Improvement in Chronic Wound Healing Using Silver Nanoparticle Hydrogel Treatment

Hanif Haidari[a, b], *Richard Bright*[c], *Sanjay Garg*[a], *Krasimir Vasilev*[c,b]*, *Allison J. Cowin*[b, a]*, *and Zlatko Kopecki*[b,a] *

[a]Clinical & Health Sciences, University of South Australia, Adelaide, SA 5000, Australia

[b]Future Industries Institute, University of South Australia, Mawson Lakes, SA 5095, Australia

[c]Academic Unit of STEM, University of South Australia, Mawson Lakes, SA 5095, Australia

Keywords: Wound infection, antibiofilm hydrogel, AgNP hydrogel, stimuli responsive hydrogel, wound healing, controlled release, and wound biofilm eradication, mature biofilm.

Table S1: Pluronic F-127 hydrogel formulation composition

Ingredients	% W/W
**Pluronic F- 127**	19
**Propylene glycol**	10
**Silver nanoparticles solution**	200 µg/g
**MilliQ water**	71

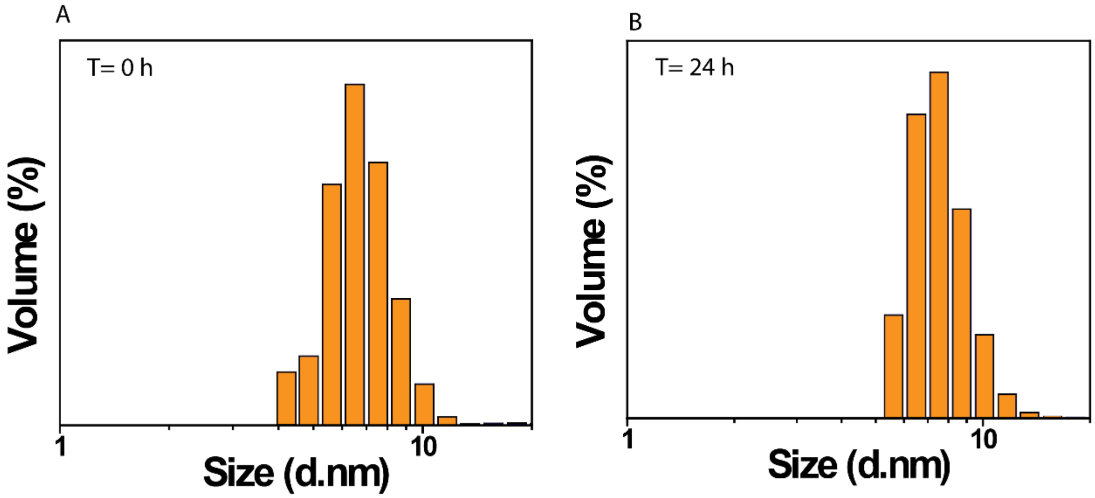

Figure S1. Dynamic light scattering (DLS) size distribution of AgNP hydrogel is measured as volume (%). (a,b) Mean hydrodynamic diameter of AgNP hydrogel in DMEM (10% FCS) at t= 0. and t=24 at 37°C storage.

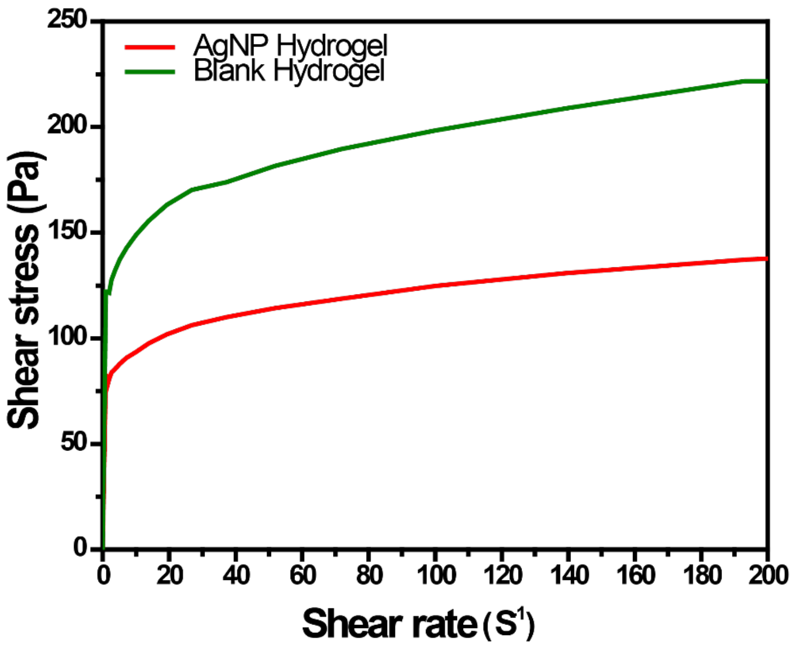

Figure S2. Measurement of shear stress in response to shear rate of pluronic f-127 hydrogel at ambient temperature.

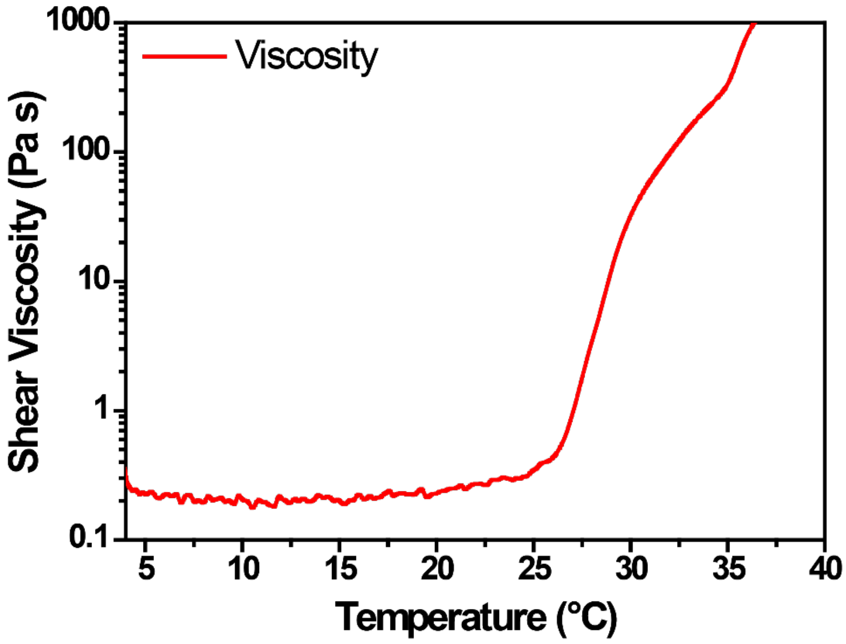

Figure S3. Measurement of shear stress in response to shear rate of pluronic f-127 hydrogel at ambient temperature.

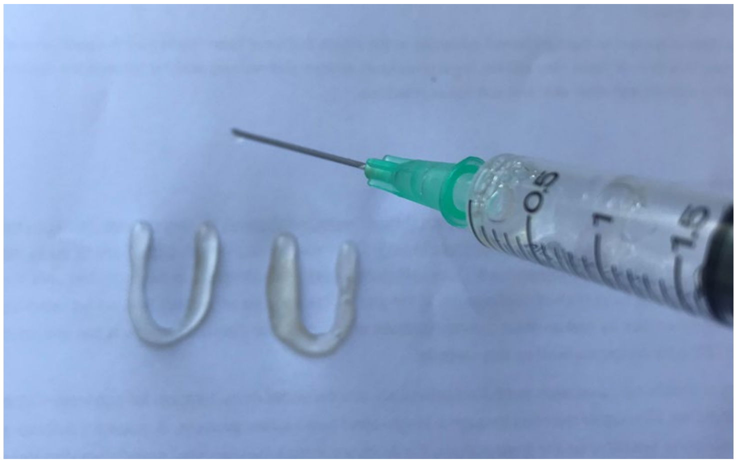

Figure S4. Photograph of AgNP hydrogel delivered using a syringe demonstraing ease of application and excellent hydrogel viscoelastic properties.

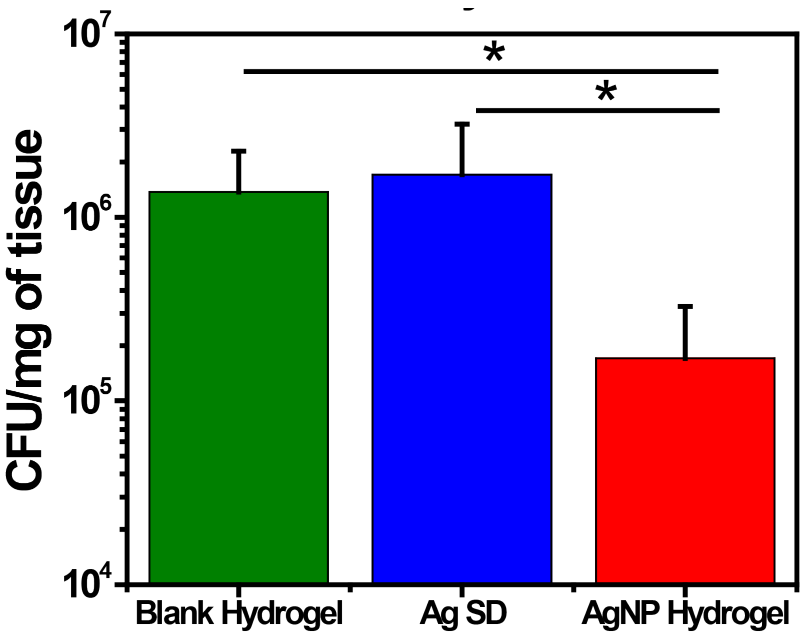

Figure S5. CFU counts of residual bacteria within wound tissue after 10 days of treatment. Data are shown as mean ±SD and the * denotes significant difference using one-way ANOVA followed by Dunnett's multiple comparison test. *$P < 0.05$, n=8.
